# Cryo-EM structures of ALECT2 filaments from human renal biopsies

**DOI:** 10.1038/s41467-026-73602-2

**Published:** 2026-05-22

**Authors:** Jian-Lin Zheng, Yu-Xin Zheng, Kai Chen, Shuang Wang, Jia-Wei Liang, Su-Xia Wang, Li Yang, Yang Shi

**Affiliations:** 1 Department of Pathology of the First Affiliated Hospital and School of Brain Science and Brain Medicine, Zhejiang University School of Medicine, Hangzhou, China; 2 Liangzhu Laboratory, MOE Frontier Science Center for Brain Science and Brain-machine Integration, State Key Laboratory of Brain-machine Intelligence, Zhejiang University, Hangzhou, China; 3 NHC and CAMS Key Laboratory of Medical Neurobiology, Zhejiang University, Hangzhou, China; 4 Laboratory of Electron Microscopy, Pathological Center, Peking University First Hospital, Beijing, China; 5 Renal Division, Renal Pathological Center, Peking University First Hospital, Beijing, China

**Keywords:** Diagnostic markers, Cryoelectron microscopy, Interstitial nephritis, Structural biology, Biophysics

## Abstract

Leukocyte chemotactic factor 2 is a recently identified amyloidogenic protein, whose abnormal aggregation defines a systemic amyloidosis termed ALECT2 amyloidosis. Due to the lack of reliable biomarkers, diagnosis relies primarily on histological demonstration and typing of amyloid deposits in renal biopsies. However, immunohistochemical detection of ALECT2 is often inconsistent, leading to diagnostic uncertainty. The underlying basis remains poorly understood, reflecting our limited knowledge of ALECT2 deposits. Here, using cryo-electron microscopy (cryo-EM), we determined the structures of ALECT2 filaments from renal biopsies of five living patients. Unlike filaments assembled from recombinant proteins in vitro, all 133 residues of mature LECT2 are incorporated into the filament cores, with native disulfide linkages preserved. The filaments consistently adopt the shared six-layered folds in all five patients, indicating a common mechanism of amyloidogenesis. Because all residues are incorporated into the fibril core, epitope accessibility is limited. This can explain variability in immunohistochemical detection and thus highlights the need for conformation-specific antibodies and antibody-independent detection strategies for improving diagnostic accuracy. This biopsy-based workflow not only expands the availability of patient-derived tissue for cryo-EM studies but also demonstrates the potential of cryo-EM as a tool for precise diagnosis of systemic amyloidosis.

## Abstract (web summary)

ALECT2 amyloidosis is defined by aggregation of LECT2. Here, the authors use cryo-EM to determine ALECT2 fibril structures from biopsies of 5 cases, showing common folds with all residues in the filament core, explaining poor epitope accessibility and enabling structure-based diagnosis.

## Introduction

Leukocyte chemotactic factor 2 amyloidosis (ALECT2) is a renal-predominant disease characterized by diffuse interstitial amyloid deposits formed by LECT2. It was described by Benson et al. in 2008 in a patient who presented with nephrotic syndrome and progressed to end-stage renal disease (ESRD) 7 years later^1^. ALECT2 amyloidosis is considered the third most common type of renal amyloidosis in the United States^2,3^, where it predominantly affects Hispanic patients of Mexican descent^4,5^. Cases have also been reported in Canada^6^, India^7^, Sudan^8^, Egypt^9^, and China^10^. Because of its mild clinical manifestations, lack of family history, rarity of extrarenal involvement, and variable degree of proteinuria, ALECT2 amyloidosis is likely to be underrecognized in clinical practice^3^. Due to the lack of reliable biomarkers, its diagnosis is based primarily on kidney biopsy. However, immunohistochemical detection of ALECT2 is inconsistent, with both false-positive^11^ and false-negative^9^ results reported.

LECT2 is mainly synthesized by the liver^12^ and is also expressed in several other organs^13^. Nascent LECT2 consists of 151 amino acids with an 18-residue N-terminal signal peptide that is cleaved prior to secretion into the circulation. Native LECT2 adopts an M23 metalloendopeptidase fold with a conserved Zn^2+^-binding site, in which Zn^2+^ is coordinated by H35, D39, H120, and a water molecule^14^. Six evolutionarily conserved cysteine residues form three intramolecular disulfide bonds (C7–C42, C18–C23, and C81–C124)^14,15^. Despite adopting a metalloendopeptidase fold, LECT2 lacks peptidase activity but plays diverse roles in chemotaxis^16^, liver regeneration^17^, skeletal growth regulation^18^, glucose and lipid metabolism^19^, and inflammation^20^.

The specific mechanism underlying LECT2 amyloidogenesis remains unclear. The binding of Zn^2+^ is considered critical for maintaining LECT2 stability in its native state, with evidence showing that in vitro amyloid filament formation is facilitated either under acidic conditions, which disrupt histidine–Zn^2+^ coordination, or under Zn^2+^-depleted conditions^21,22^. In addition, nearly all patients have been reported to be homozygous for the G-allele (SNP rs31517, exon 3) at nucleotide 172 of the *LECT2* gene, leading to a substitution of isoleucine with valine at position 40 (I40V)^1,23,24^, but the mechanism underlying this correlation remains unresolved.

Cryogenic electron microscopy (cryo-EM) has enabled the structure determination of amyloid filaments from patient tissue^25–30^, which, to date, have been predominantly derived from post-mortem samples. Structural polymorphism enables filaments to adopt different folds in distinct pathological environments^26^, thereby incorporating pathological information. Beyond the ordered filament core, amyloids often contain less well-ordered sequences flanking the amyloid core that form a structurally disordered “fuzzy coat,” which may influence the spreading and toxicity of the assemblies^31^.

Here, we present the cryo-EM structures of ALECT2 filaments extracted from renal biopsies of five individuals with ALECT2 amyloidosis. The shared six-layered ALECT2 protofilament folds, lacking a fuzzy coat, were observed in all patients and are distinct from those formed in vitro^22^, suggesting a common amyloidogenesis mechanism in ALECT2 amyloidosis.

## Results

### Pathological characterizations

**Fig. 1:**
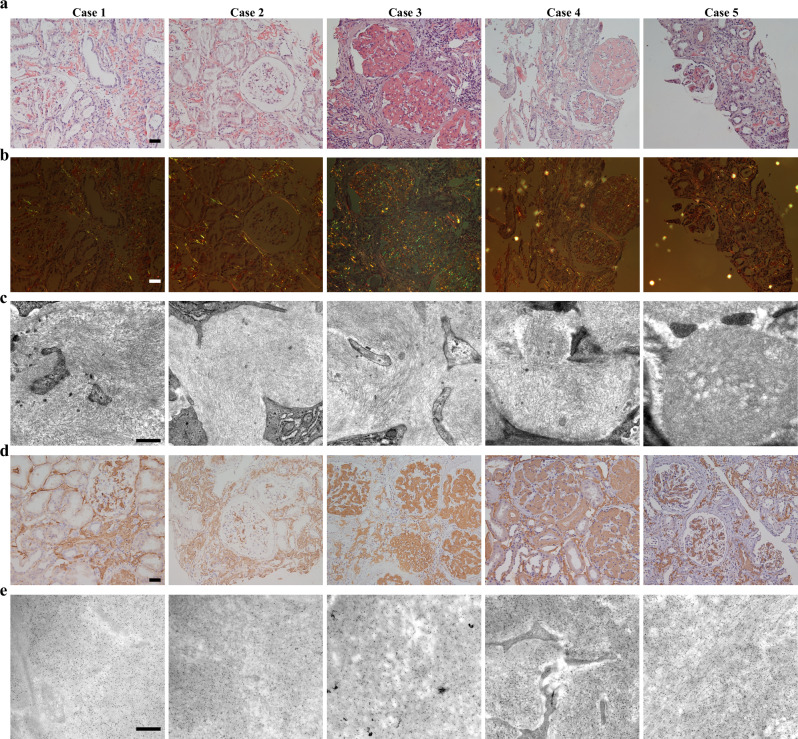
Filamentous ALECT2 pathology from ALECT2 amyloidosis patients. **a, b** Congo red-stained sections of glomeruli and cortical interstitium (**a**), showing apple-green birefringence under polarized light (**b**). **c** Resin-embedded tissue sections imaged by electron microscopy, revealing the fibrillar nature of deposits. **d, e** Immunohistochemistry (**d**) and immuno-electron microscopy (**e**) labeling of LECT2 deposits with anti-LECT2 antibody. Scale bar: 50 µm (**a, b, d**), 500 nm (**c, e**). All experiments in **a**–**e** were independently repeated three times with similar results.

Renal biopsy specimens for cryo-EM analysis were obtained from five patients with ALECT2 amyloidosis. All patients presented elevated serum creatinine and reduced estimated glomerular filtration rate (eGFR), indicating varying degrees of renal dysfunction (Supplementary Table 1). Congo red–positive deposits that gave green birefringence when viewed with polarized light were detected in all cases (Fig. 1a, b). Electron microscopy (EM) revealed the filamentous aggregates within the deposits (Fig. 1c), and immunohistochemistry and immuno-EM with an anti-LECT2 antibody confirmed that these filamentous assemblies were composed of LECT2 (Fig. 1d, e). Genetic analysis for exon 3 of *LECT2* gene revealed that all patients were homozygous for the G allele at nucleotide 172 (SNP rs31517, c.172A>G), resulting in substitution of isoleucine with valine at residue 40 of the mature protein (Supplementary Fig. 1a).

### Extraction of ALECT2 filaments for cryo-EM

We extracted ALECT2 filaments from <10 mg renal biopsies obtained from each individual, using a modified water-extraction protocol as described previously^32^. Immunoblotting with a human LECT2 polyclonal antibody detected a ~16 kDa band corresponding to full-length monomeric LECT2, and additional bands at ~32 kDa and higher molecular weights, corresponding to dimeric and higher-order ALECT2 assemblies resistant to SDS denaturation (Supplementary Fig. 1b).

**Fig. 2:**
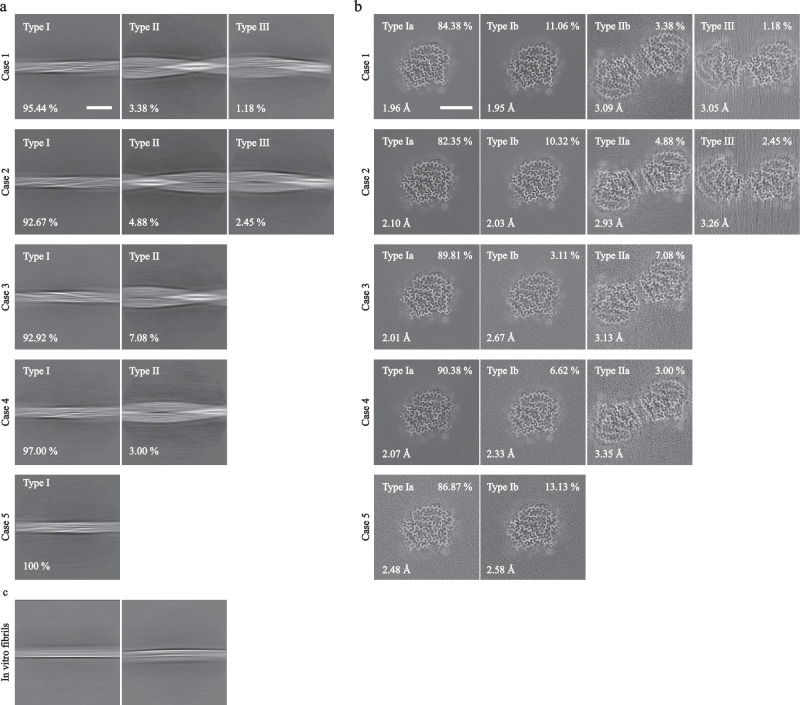
Cryo-EM analysis of ALECT2 filaments. **a, c** Representative 2D class averages of patient-derived (**a**) and in vitro ALECT2 filaments (**c**). Scale bar, 20 nm. **b** Cross-sections of ALECT2 filaments from five cases, perpendicular to the helical axis and with a projected thickness of approximately one β-rung. Cryo-EM data for each case were collected and processed once. Filament types, percentages of each filament type, and resolution (in Å) are indicated at the top left, top right, and bottom left, respectively. Scale bar, 5 nm.

Filaments extracted from the renal biopsy tissue of each patient were sufficient for cryo-EM analysis, enabling the determination of 16 ALECT2 filament structures at resolutions of 1.9–3.4 Å (Fig. 2; Supplementary Fig. 2; Supplementary Table 2). Three filament types (I–III) were identified: type I consisted of a single protofilament, whereas types II and III each comprised two protofilaments, with symmetric packing in type II and asymmetric packing in type III. Type I filaments were the predominant species in all five patients. Single- and double-protofilament arrangements were occasionally observed to coexist within the same filaments (Supplementary Fig. 1c).

To assess whether these patient-derived ALECT2 filaments are seeding competent, we next evaluated their seeding activity in vitro. Biopsy-extracted ALECT2 filaments were mixed with recombinant monomeric LECT2 at 10% (w/w), and fibril formation was monitored by thioflavin T (ThT) fluorescence at 37°C under constant orbital shaking. To facilitate the unfolding of recombinant monomeric LECT2, the assay was performed at pH 4.4. Compared with reactions without seeds, the addition of the patient-derived seeds shortened the lag phase (the initial time required for fibril formation), indicating that the biopsy-extracted ALECT2 filaments accelerate the fibril formation of recombinant LECT2 in vitro (Supplementary Fig. 1d). The formation of filamentous assemblies was confirmed by negative-stain EM (Supplementary Fig. 1e).

### Protofilament structures

**Fig. 3:**
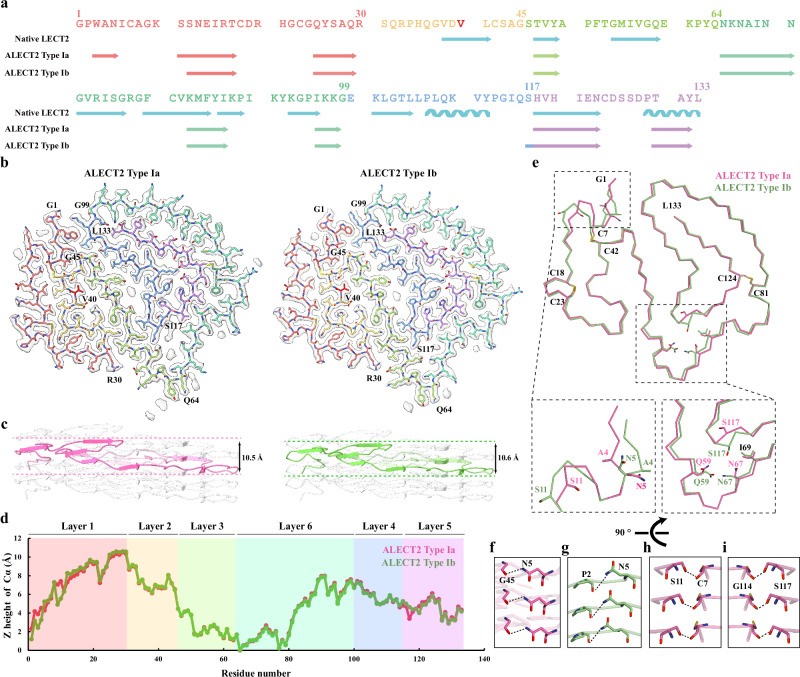
Two ALECT2 protofilament folds from human renal biopsies. **a** Amino-acid sequence of the mature LECT2-V40 variant and the secondary structure elements in its native, filament Ia, and filament Ib folds (β-strands shown as arrows, and α-helices as curved lines). **b** Cryo-EM density maps (transparent gray) and atomic models for type Ia (left) and Ib (right) filaments. The six layers are defined as follows: layer 1, residues 1–30; layer 2, residues 31–45; layer 3, residues 46–64; layer 4, residues 100–117; layer 5, residues 118–133; and layer 6, residues 65–99. **c** Views perpendicular to the helical axis showing differences in height along the helical axis within a single LECT2 molecule in Ia (left) and Ib (right) folds. **d** Cα height profiles along the helical axis (Z) for a single LECT2 molecule in Ia (hot pink) and Ib (lime) folds. **e** Structural superposition of Ia and Ib folds. Dashed boxes indicate structural differences between the two folds, which are realigned in the zoom-in views based on local structural similarities. **f–i** Views perpendicular to the helical axis showing side chains of residues N5 (**f, g**), S11 (**h**), and S117 (**i**) in the Ia fold forming hydrogen bonds with main chains. The Ia fold is shown in hot pink and the Ib fold in lime. Source data are provided as a Source Data file.

Further three-dimensional classification revealed two subtypes of type I filaments (Ia and Ib), both of which were present in all five patients, with Ia consistently predominating (Fig. 2). In addition, both type II and type III filaments were formed from identical protofilaments that adopt the Ia fold. Structure determination at resolutions sufficient for de novo atomic modeling revealed that the ordered cores of all filaments include the entire mature protein, spanning residues 1–133 (Fig. 3a, b and Supplementary Fig. 3a). Notably, extracted filaments showed weaker immunogold labeling in negative-stain EM (Supplementary Fig. 1f) than in tissue sections (Fig. 1), despite the use of the same primary antibody (AF722). A similar binding behavior has been reported for core-tau antibodies^33^, suggesting that the epitope recognized by anti-LECT2 antibody AF722 is buried within the filament core, with antigen retrieval potentially facilitating its exposure during immunohistochemistry^34^.

Both the Ia and Ib folds share a similar six-layered ordered core, with a backbone root mean square deviation (RMSD) of 1.027 Å between them (Supplementary Table 3). The Ia fold contains nine short β-strands, and the Ib fold contains eight, each spanning between 3 and 7 residues (Fig. 3a and Supplementary Fig. 3a). Residues G1–G45 in the N-terminal region form the first two layers of the six-layered ordered core, adjacent to the third layer formed by residues S46–Q64. In the C-terminal region, residues E100–L133 form the fourth and fifth layers, which are surrounded by the sixth layer formed by residues N65–G99 in the middle region (Fig. 3b).

In both subtypes, the maximum height difference of a single molecule along the helical axis is about 10.5 Å, observed between layer 2 and layer 3, resulting in inter-rung interactions that contribute to filament stabilization (Fig. 3c, d). In addition, the Zn^2+^-binding site observed in the native LECT2^14^ is not present in the filaments, with H120 located far from H35 and D39 (Fig. 3b).

The structural differences between the Ia and Ib folds are found in layer 1 and the turn between layers 4 and 5 (Fig. 3e). In layer 1, the side chains of A4, N5, and S11 adopt different orientations between the two folds. In the Ia fold, the side chains of N5 and S11 form hydrogen bonds with the main-chain carbonyl oxygens of G45 and C7, respectively. By contrast, in the Ib fold, the side chain of N5 interacts with the main-chain carbonyl oxygen of P2, and the side chain of S11 is solvent-exposed (Fig. 3f–h). At the turn between layers 4 and 5, S117, which forms a hydrogen bond with the backbone carbonyl group of G114 in the Ia fold, is flipped in the Ib fold and oriented toward a cavity (Fig. 3i). These two local conformational changes make the Ia fold more compact, with a low solvation energy (−66.6 kcal/mol, per rung) compared to the Ib fold (−66.2 kcal/mol, per rung) (Supplementary Fig. 3b).

### Buried polar interactions

ALECT2 filaments are intricately stabilized by numerous polar interactions within the filament core (Supplementary Fig. 3a, c). In the Ia fold, the side chains of residues N5, S11, N13, Y26, S31, Q32, Q36, S43, Q109, and S117, most of which are located within layers 1 and 2, form hydrogen bonds with main-chain carbonyl groups. Notably, the hydrogen bonds involving S11 and S117 are absent in the Ib fold, distinguishing the Ia fold from the Ib fold (Fig. 3e and Supplementary Fig. 3c).

In addition, four clusters of buried charged residues were observed in ALECT2 filaments (Supplementary Fig. 3c). Among them, R33–D39 and K88–D128 form two salt bridges that neutralize the buried charges and stabilize the filament structure. However, the other two clusters, K110–E122–D125 and K97–E100–L133–COOH, each contain a salt bridge and an uncomplemented carboxyl group. These buried, uncomplemented negative charges raise the energetic barrier to filament nucleation, but this barrier is reduced under acidic conditions, providing a possible clue to the environmental conditions favoring ALECT2 filament formation.

### Disulfide bonds

The cryo-EM maps are of sufficient quality to allow identification of the disulfide bonds in patient-derived ALECT2 filaments. Both protofilament folds contain the same three intramolecular disulfide bonds: C7–C42, C18–C23, and C81–C124, consistent with those identified in recombinant native LECT2 in vitro^14,15^ (Supplementary Fig. 4a). The disulfide bonds C7–C42 and C81–C124, which connect layers 1 and 2 and layers 5 and 6 in the fibrillar state, adopt markedly different local conformations compared with the native structure, with their linked segments extending in different directions (Supplementary Fig. 4b). The disulfide bond C18–C23 is located within the first layer in the fibrillar state, with the linked segments extending in the same direction in both native and fibrillar structures, whereas the residues flanking the disulfide loops undergo substantial structural rearrangements (Supplementary Fig. 4b). Together, these observations suggest that the local structures surrounding the disulfide bonds are not preserved during the transition from the native to the fibrillar state, highlighting an important role of disulfide bonds in the filament folds.

The recombinant LECT2 (I40V) used for seeding assays and in vitro filament assembly was refolded under non-reducing conditions to allow reformation of disulfide bonds. SDS-PAGE showed a single band under both reducing and non-reducing conditions, with non-reduced LECT2 migrating faster than the reduced form (Supplementary Fig. 4c). This is consistent with a previous study^35^ and suggests that the refolded LECT2 adopted a compact monomeric structure with intramolecular disulfide bonds. Mass spectrometry analysis further confirmed the presence of three disulfide linkages within the protein (Supplementary Fig. 4d, e), although both native and non-native disulfide pairings were detected (Supplementary Table 4).

However, the filaments formed by refolded LECT2 lacked an apparent helical twist and were therefore not structurally determined by cryo-EM (Fig. 2c). In addition, these filaments exhibited antibody-binding properties distinct from those of patient-derived filaments. Using the same primary antibody, they were labeled with immunogolds in negative-stain EM (Supplementary Fig. 1g) but lost this labeling after proteinase K (PK) digestion (Supplementary Fig. 1h). The presence of the PK-sensitive epitope in the in vitro–formed filaments suggests that their filament core is distinct from that of patient-derived filaments.

### Cavities and additional densities

Like other patient-derived amyloid filaments^25–30^, non-proteinaceous additional densities were also observed in ALECT2 filaments. In both Ia and Ib folds, cavities formed by the side chains of Y49, P51, T53, P113, and L108 are structurally identical and accommodate extra densities with similar shapes (Supplementary Fig. 5a). In contrast, cavities formed by residues Q116, Q59, N67, and I69 differ between the two folds, with the cavity in the Ia fold enlarged due to flipped S117 and side-chain conformational changes of Q59 and N67 (Fig. 3e). The extra densities within these cavities also differ: in the Ia fold, two distinct densities are observed, whereas in the Ib fold, a single water-like density occupies the side-chain groove between N67 and I69 (Supplementary Fig. 5b). Similarly, the grooves formed by residues P2, A4, G9, and K10 also differ between the two folds due to distinct side-chain orientations of A4, N5, and S11, with the groove in the Ia fold being more compact (Fig. 3e). Correspondingly, the additional densities are in different shapes: an ellipsoidal column with poor axial continuity in the Ia fold, and a more continuous cylindrical form in the Ib fold (Supplementary Fig. 5c). In type Ib filaments, the flipped S11 also prevents binding of an extra density in the groove formed by K10 and S12 that is present in the Ia fold (Supplementary Fig. 5c). Together, these observations suggest that local conformational changes in the cavities alter the binding or sequestration profiles of non-proteinaceous molecules, potentially resulting in different impacts on the surrounding environment among filament polymorphs.

### Residue 40

Genetic sequencing revealed that all five patients in this study were homozygous for the G allele at nucleotide 172, encoding the LECT2-V40 variant. This result is consistent with the high-resolution side-chain density at residue 40, which corresponds more closely to valine than isoleucine (Fig. 3b). Residue V40 forms hydrophobic contacts with V48 at the interface between layers 2 and 3, with the closest interatomic distance of 4.3 Å between their side-chain carbon atoms. However, substituting valine with isoleucine reduces this distance to below the sum of the van der Waals radii of two carbon atoms (3.4 Å), thereby introducing steric hindrance (Supplementary Fig. 6). These observations suggest that the space around residue 40 is unlikely to accommodate an isoleucine substitution without altering the filament structure, providing a structural explanation for the association of the LECT2-V40 variant with disease. As reproducing the same fold with recombinant proteins in vitro is challenging^22^, this structural evidence can hardly be observed in vitro.

### Dimeric filaments

**Fig. 4:**
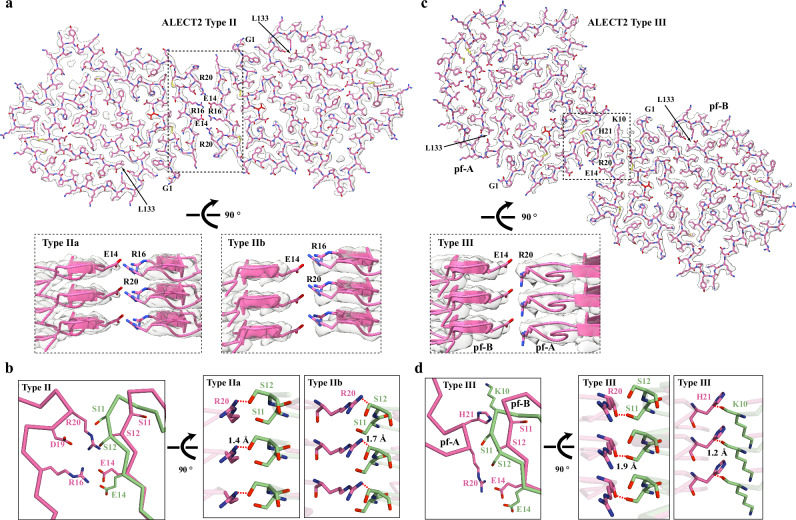
Structures of ALECT2 type II and III filaments. **a, c** Cryo-EM density maps (transparent gray) and atomic models of ALECT2 type II fold (**a**) and III fold (**c**). For type II filaments, IIa and IIb folds are identical in the cross-section view (top) but differ in protofilament packing along the helical axis (bottom). Type IIa adopts C2 symmetry, whereas type IIb shows approximate 2_1_ screw symmetry. **b, d** Close-up views of the protofilament interfaces of type II (**b**) and type III (**d**) filaments, overlaid with the type Ib filament (lime). Red dashed lines indicate the closest atomic contacts, highlighting potential steric clashes if type Ib filaments were incorporated into type II or III assemblies.

Type II filaments were observed in cases 1–4, which are characterized by two symmetrically packed protofilaments. Although their cross-sectional views appear nearly identical, the helical symmetries vary among patients. In cases 2–4, the two protofilaments are arranged with a C2 symmetry (type IIa; Fig. 4a), whereas, in case 1, they adopt an approximate 2_1_ screw symmetry (type IIb; Fig. 4a). The buried surface area per rung at the protofilament interface is 180.67 Å^2^ for type IIa and 223.04 Å^2^ for type IIb. The larger buried surface area observed in the 2₁ screw–packed filaments indicates that a half-rise offset between the two protofilaments increases the inter-protofilament interaction. At the protofilament interface, two salt bridges between R16 and E14 from opposing protofilaments stabilize the inter-protofilament packing in both subtypes. Notably, incorporating the Ib fold into type II filaments would create steric clashes between the outward-shifted S12 and R20 from the neighboring protofilament, which may explain why the Ib fold is absent in these filaments (Fig. 4b).

Type III filaments were observed in cases 1 and 2, consisting of two asymmetrically packed protofilaments (pf-A and pf-B). Molecules from opposing protofilaments are positioned at the same height along the helical axis, resembling the arrangement in type IIa filaments (Fig. 4c). The buried surface area per rung at the protofilament interface is 173.32 Å^2^. The protofilament interface is stabilized by a salt bridge between R20 (pf-A) and E14 (pf-B), together with a hydrogen bond between H21 (pf-A) and the backbone carbonyl oxygen of K10 (pf-B), in contrast to type II filaments, in which the interface is mediated exclusively by side-chain–side-chain electrostatic interactions. As in type II filaments, this inter-protofilament interaction is likely disrupted by the outward flip of S11 in the Ib fold, which introduces steric hindrance with R20 and may explain the absence of the Ib fold in pf-B of type III filaments (Fig. 4d). In addition, the predominance of type Ia filaments, together with the low proportion of dimeric filaments (Fig. 2), may also explain why only dimeric filaments incorporating the Ia fold were observed.

## Discussion

In this study, we determined filament structures from renal biopsy samples of five individuals with ALECT2 amyloidosis. Both unpaired and paired filaments were observed, with unpaired filaments predominating, and all shared common protofilament folds. Consistent with our observations, a recent study showed the presence of both paired and unpaired filaments with similar relative abundances in a nephrectomy specimen from a patient with ALECT2 amyloidosis^36^, suggesting a conserved ratio among these patients. However, type Ib filament, a minor subtype in our study, is the predominant filament type in the nephrectomy specimen. The differential predominance of protofilament subtypes among patients may warrant further investigation.

In the tightly packed six-layered ALECT2 filament, residues within a single molecule are positioned at different heights along the helical axis, allowing inter-layer interactions to contribute not only to intra-molecular stabilization but also to inter-molecular interactions. Representative examples of the latter include the side-chain–backbone interaction between Q32 and M55 at the interface between the second and the third layers, as well as the Y92–Y132 interaction at the interface between the fifth and the sixth layers (Supplementary Fig. 3d). Additional contributions to axial stabilization arise from extended hydrogen-bonding networks formed by glutamine and asparagine residues, as well as staggered stacking interactions between aromatic rings (Supplementary Fig. 3d). Together, these interactions cooperatively stabilize the filament along the helical axis.

Distinct from intrinsically disordered or low-complexity amyloid proteins, LECT2 adopts the M23 metalloendopeptidase fold in its native state, indicating that denaturation of this native fold is a prerequisite for subsequent filament formation. Acidic pH is a well-established trigger of filament formation in vitro by destabilizing the proteins in their native states^37–39^, and this may also apply to LECT2^22^. Protonation of histidine residues at acidic pH abolishes Zn^2+^ binding, thereby destabilizing the native LECT2 fold, constituting a LECT2-specific pH response mechanism that may make LECT2 more sensitive to acidic conditions^21^. The uncompensated buried carboxyl groups were observed in the regions of E122–E125–K110 and K97–E100–L133–COOH, which is unusual among human tissue-derived amyloids, as electrostatic repulsion between adjacent rungs would increase the energetic cost of filament formation. Four uncompensated buried acidic residues were observed in patient-derived TMEM106B filaments, which were proposed to be protonated under acidic lysosomal conditions, thereby overcoming the energetic barrier to fibril nucleation^40^. However, the presence of multiple buried salt bridges (R33–D39 and K88–D128, K110–E122–D125 and K97–E100–L133–COOH) in ALECT2 filaments suggests that filament formation requires a selective protonation mechanism. In addition, the *pKₐ* values of buried, uncompensated carboxyl groups are known to be substantially elevated relative to their solvent-exposed counterparts, whereas those of buried carboxyl groups forming salt bridges remain near their aqueous *pKₐ* values^41^. Solid-state NMR studies have shown that an uncompensated buried glutamic acid in β-endorphin filaments remains protonated even at pH 7.4^42^. We therefore propose a coupled filament nucleation–carboxyl protonation model: solvent-exposed carboxyl groups with low *pKₐ* values remain unprotonated at the onset of nucleation, enabling salt-bridge formation. As filament assembly proceeds and some of the carboxyl groups become buried within a low-dielectric environment, those stabilized by salt bridges remain largely unaffected, while the *pKₐ* values of uncompensated carboxyl groups increase, favoring their protonation even in the absence of strongly acidic conditions. Taken together, although there is currently no direct evidence that ALECT2 formation in vivo depends on an acidic environment, acidic pH may facilitate both the denaturation of native LECT2 (histidine protonation) and the subsequent formation of ALECT2 filaments (carboxyl protonation).

Although LECT2 undergoes substantial structural changes from its native to fibrillar state, the disulfide linkage pattern is unchanged^14^, suggesting that the six cysteines retain the disulfide linkages during filament formation. A previous study showed that, in contrast to patient-derived ALECT2 filaments, only residues M55–I75 were incorporated into the cores of filaments formed by recombinant full-length LECT2 under reducing conditions (30 mM TCEP)^22^, resembling part of corresponding region (I56–N67) in patient-derived filaments (RMSD = 0.762 Å) (Supplementary Fig. 7 and Supplementary Table 3). However, the core of in vitro–formed filaments lacks cysteine residues incorporation and is confined to the region between the two disulfide bonds (C7–C42 and C81–C124; Supplementary Fig. 7), suggesting that disulfide bonds may affect the aggregation propensity of surrounding residues. Moreover, within the G1–S12 region where the Ia and Ib folds differ, the local conformations at residue C7 (disulfide-bonded to C42) are nearly identical in both folds (Fig. 3e), suggesting that the disulfide linkages constrain structural polymorphism. Consistently, studies on insulin have shown that reducing conditions favor the formation of heterogeneous filaments^43^. Together, these observations indicate that the disulfide bonds play an important role in determining the filament folds.

Amyloid typing has traditionally relied on immunohistochemistry using antibodies that target amyloid deposits. Because flexible fuzzy coats are often accessible to antibodies, most deposit-targeting antibodies recognize epitopes located in these regions^44,45^. However, our structural analysis reveals that LECT2 undergoes a near-complete conversion from its native fold into a compact filament core, without leaving residues to form a fuzzy coat. Like other core-specific antibodies^33^, we found that the anti-LECT2 antibody AF722 shows limited binding to ALECT2 filaments in negative-stain EM but exhibits labeling in immunohistochemistry, possibly due to enhanced epitope accessibility following antigen retrieval^34^. The limited epitope accessibility may compromise the reliability of immunohistochemical detection of ALECT2^9,11^, highlighting the need to develop conformation-specific antibodies and antibody-independent approaches to improve the diagnosis of ALECT2 amyloidosis.

In this study, we established a biopsy-based cryo-EM workflow that enables structural determination of ALECT2 filaments from <10 mg of renal biopsy tissue, achieving resolutions comparable to those obtained from post-mortem samples. This workflow is applicable to biopsy specimens containing abundant amyloid filaments that exhibit an apparent helical twist. Accurate amyloid typing is essential for prognosis, therapy, and genetic counseling in renal amyloidosis. Misinterpretation may lead to serious consequences^46^. Cryo-EM structure determination has previously been shown not only to identify previously unknown filaments^40,47,48^, but also to classify diseases based on their filament folds^26^. By showing that high-resolution cryo-EM structures can be obtained from minimal biopsy materials, our study demonstrates the potential of cryo-EM as a precise diagnostic tool for systemic amyloidosis.

## Methods

### Ethics statement

The collection and use of human renal biopsy specimens in this study were approved by the Ethics Committee of Peking University First Hospital (approval number 2022[448-002]). Written informed consent was obtained from all patients.

### Source of ALECT2 filaments

Renal tissues obtained from five patients during biopsy (cases 1–5) were preserved in optimal cutting temperature (OCT) compound and frozen at −80°C. No additional sampling procedures were performed beyond standard clinical requirements. Cases 4 and 5 were previously reported as patients 3 and 6, respectively^49^. Detailed clinicopathological information is provided in Supplementary Table 1.

### Genotyping of the rs31517 variant (I40V)

DNA was extracted from peripheral blood as previously described^50^. Exon 3 of the *LECT2* gene was amplified by polymerase chain reaction (PCR) using the primers (BerryGenomics) 5′-tacatggactgcctcat (forward) and 5′-tttctgttccaggtttt (reverse), yielding a 574-bp fragment. The PCR product was then subjected to Sanger sequencing, and the resulting data were analyzed using SnapGene (version 7.0.2) and compared with the Human Genome reference sequence (GRCh38).

### Extraction of ALECT2 filaments

LECT2 filaments were extracted from frozen renal biopsies using a previously described water-extraction protocol^32^ with minor modifications. Briefly, <10 mg of tissue was pelleted by centrifugation (3100 × *g*, 10 min, room temperature), and the supernatant was discarded. The pellet was washed three times with Tris-calcium buffer (20 mM Tris-HCl, 138 mM NaCl, 2 mM CaCl₂, pH 8.0), each wash consisting of gentle resuspension followed by centrifugation at 3100 × *g* for 10 min. The washed tissue was then resuspended in Tris-calcium buffer and incubated with 5 mg/mL collagenase (V900893; Sigma) at 37°C for 3 h with gentle agitation. After incubation, the suspension was centrifuged at 3100 × *g* for 10 min at 4°C. The pellet was resuspended in ice-cold water and subjected to non-contact bath sonication at 900 W (5 s on/5 s off duty cycle) for 5 min (SCIENTZ08-IIIC, Ningbo Scientz Biotechnology Co., China). Large tissue debris was collected by centrifugation (3100 × *g*, 10 min, 4°C), and subjected to additional rounds of extraction until no visible pellet was observed. All supernatants containing ALECT2 filaments were collected and pooled for cryo-EM analysis. For seeding assays, samples were further purified by treatment with 1% (w/v) sarkosyl, followed by ultracentrifugation at 112,000 × *g* for 1 h using a TLA-55 rotor (Beckman Coulter). The supernatant was discarded, and the resulting pellet was resuspended in water.

### Expression and purification of LECT2

The cDNA encoding mature, full-length human LECT2 carrying the I40V single-nucleotide polymorphism was synthesized and cloned into the pET-21a vector (Hangzhou Youkang Biotechnology Co., Ltd., Hangzhou, China). The plasmid was transformed into *E. coli* BL21(DE3) (11804ES, Yeasen) for expression. LECT2 was purified from inclusion bodies. Briefly, cell pellets expressing LECT2 were resuspended in Buffer A (50 mM Tris-HCl, pH 8.0, 150 mM NaCl, 1 mM phenylmethylsulfonyl fluoride (PMSF)), followed by sonication and centrifugation to collect the insoluble fraction. The inclusion-body pellet was washed three times with Buffer A containing 2% (v/v) Triton X-100, then dissolved in Buffer A supplemented with 6 M guanidine hydrochloride and 10 mM dithiothreitol (DTT) overnight at 4°C. Solubilized material was dialyzed against buffer containing a reduced glutathione (GSH)/oxidized glutathione (GSSG) redox pair, and the protein was further purified by cation-exchange chromatography followed by size-exclusion chromatography (Superdex 75, Cytiva).

### Amyloid seeding assays

The fibril extracts were subjected to probe sonication to generate short fibril seeds (3 s on, 3 s off, total sonication time of 2 min; JY92-IIN, SCIENTZ). The concentration of fibril seeds was determined using BCA Protein Assay Kit (Beyotime). Seeds were added at 10% (w/w) to 25 μM recombinant LECT2 (I40V) in a total volume of 100 μL. Reactions were carried out in 50 mM phosphate buffer (pH 4.4) containing 150 mM NaCl and 5 μM thioflavin T (ThT). Plates were incubated at 37°C with continuous shaking at 900 rpm, and ThT fluorescence was measured every 10 min (excitation 440 nm, emission 482 nm) using a FLUOstar Omega microplate reader (BMG LabTech). All experiments were performed in triplicate.

### Mass spectrometry

The intact mass of refolded LECT2-I40V under non-reducing (*n* = 1) and reducing conditions (*n* = 1) was analyzed by liquid chromatography–electrospray ionization–time-of-flight mass spectrometry (LC-ESI-TOF-MS). For sample preparation, aliquots (0.5 mg/mL) were denatured in 8 M urea, 50 mM HEPES (pH 6.5) for 1 h at room temperature. Aliquots designated for reduction were subsequently treated with 5 mM tris(2-carboxyethyl)phosphine (TCEP) for 1 h at room temperature. Both reduced and non-reduced samples were then alkylated with 50 mM iodoacetamide for 20 min in the dark. Reactions were stopped by adding formic acid to a final concentration of 0.5% (v/v). Samples were subsequently buffer-exchanged into 0.1% formic acid using a centrifugal filter unit with a 3 kDa molecular weight cutoff (Merck Millipore). Chromatographic separation was performed on a UPLC system (Waters) with a BEH C4 column (50 mm × 2.1 mm, 1.7 μm) at 40°C, using a gradient from 5% to 95% acetonitrile in 0.1% formic acid at a flow rate of 0.3 mL/min. Mass spectra were acquired on a quadrupole time-of-flight mass spectrometer (Synapt XS, Waters) operated in positive-ion mode, with full MS scans collected over an *m*/*z* range of 200–2000. The data were acquired and processed using Masslynx (version 4.2).

Disulfide mapping of recombinant LECT2-I40V (*n* = 1) was performed following a previously described non-reducing LC–MS/MS workflow^51^. Samples were buffer-exchanged into UA buffer (8 M urea, 150 mM Tris-HCl, 2 mM N-ethylmaleimide, pH 6.5) and incubated at 37°C for 2 h, followed by overnight digestion with trypsin at 37°C. The resulting peptides were desalted using C18 StageTips, and 2 µg of protein digest was subjected to LC–MS/MS analysis. Chromatographic separation was performed on an EASY-nLC 1200 (Thermo Scientific) equipped with a C18 column (75 μm × 150 mm, 3 μm; Dr. Maisch GmbH) using a gradient from 2% to 80% acetonitrile in 0.1% formic acid. Mass spectra were acquired on a Q Exactive HF-X mass spectrometer (Thermo Scientific) operated in data-dependent acquisition (DDA) and positive-ion mode. The *m*/*z* range of MS1 was 350–1800 with a resolution at 60,000, with an automatic gain control (AGC) target of 3 × 10^6^, and a maximum ion injection time (max IT) of 50 ms. The top 20 most intense precursor ions carrying +2 to +6 charges were selected for fragmentation by higher-energy collisional dissociation (HCD). MS/MS spectra were acquired at a resolution of 15,000, with an AGC target of 1 × 10^5^, and a max IT of 25 ms. Raw data were analyzed with pLink (version 2.3.9) with the built-in disulfide-search mode (Cys–Cys linkages) applied to the non-reducing LC–MS/MS data. Searches were conducted against the sequence of mature human LECT2 (I40V). The search parameters were set as follows: precursor mass tolerance of 10 ppm, fragment ion tolerance of 0.02 Da, up to three missed cleavages, peptide mass range of 600–6000 Da, and peptide length of 6–60 amino acids. N-ethylmaleimide-modified cysteine was included as a variable modification, and results were filtered using a false discovery rate (FDR) threshold of 1%.

### In vitro assembly of LECT2 filaments

Purified LECT2 was diluted to 100 µM in fibrillization buffer (150 mM NaCl, pH 2.0) in a 2-mL microcentrifuge tube. Samples were incubated in a ThermoMixer (HCM100-Pro, DALB) at 37°C with orbital shaking at 1450 rpm to induce fibrillization. Fibril growth was monitored by thioflavin T (ThT) fluorescence (excitation 440 nm, emission 485 nm) at a final ThT concentration of 30 µM.

### Immunolabeling and histology

Histology, immunohistochemistry, and tissue-section immunogold labeling were carried out as previously described^49^. Congo red staining followed by hematoxylin counterstaining was carried out on 10-µm paraffin-embedded sections, immunohistochemistry on 4-µm paraffin-embedded sections, and immunogold labeling on 80-nm resin-embedded sections. For both immunohistochemistry and immunogold labeling on tissue sections and in negative-stain EM, the primary antibody against LECT2 (AF722, R&D Systems) was used at a 1:40 dilution. For immunoblots, samples were mixed with 5× SDS-PAGE loading buffer (20315ES20; Yeasen), heated at 100°C for 10 min, and resolved on 15% gels (36248ES10, Yeasen). Polyclonal anti-human LECT2 antibody (AF722, R&D Systems) was used at a 1:1000 dilution.

### Cryo-electron microscopy

Holey carbon grids (Nanodim Au R1.2/1.3, 300 mesh) were glow-discharged for 60 s using a Coolglow glow discharge system operated at 40 mA. Samples were applied to glow-discharged grids and plunge-frozen in liquid ethane using Thermo Scientific Vitrobot Mark IV System at 4°C and 100% humidity. Cryo-EM images were acquired using a 300 kV Titan Krios microscope (Thermo Fisher) equipped with a cold field-emission gun, a Falcon 4i detector, and a Selectris-X energy filter. The energy filter was operated with a slit width of 10 eV to remove inelastically scattered electrons. Aberration-free image shift in the EPU software (Thermo Fisher) was used during image acquisition. Further details are given in Supplementary Table 2.

### Helical reconstruction

Datasets were processed in RELION 5.0 using standard amyloid helical reconstruction procedures^52^. Movie frames were gain-corrected, aligned, and dose-weighted using RELION’s own motion-correction program^53^. The contrast transfer function (CTF) parameters were estimated using CTFFIND-4.1^54^. Filaments were picked manually and extracted with a box size of 900 pixels, subsequently downscaled to 300 pixels. Reference-free 2D classification was performed to remove low-quality segments, and representative 2D class averages for each filament type were selected to generate initial models using relion_helix_inimodel2d. For high-resolution refinement, selected segments from each filament type were extracted with a box size of 256 pixels at the original pixel size. 3D auto-refinements were performed, with optimization of the helical twist and rise parameters once resolutions extended beyond 4.8 Å. Bayesian polishing and CTF refinement were applied to further improve the resolution^53^. The structure heterogeneity was examined by 3D classification without alignment. Final maps were sharpened using standard postprocessing procedures in RELION, and resolution estimates were calculated based on the Fourier shell correlation (FSC) between two independently refined half-maps at 0.143^55^. Further details are given in Supplementary Table 2.

### Atomic modeling

Atomic models were built and refined in the best available maps for each filament type. For type Ia, Ib, IIb, and III filaments, the maps were from case 1; for type IIa filament, the map was from case 2. Initial models for each filament type were generated using CryoAtom^56^ (version 1.3.1), with amino-acid sequence of the LECT2-V40 variant provided as input. The models were subsequently subjected to iterative cycles of automated real-space refinements in PHENIX^57^ (version 1.21-5207-000) and manual adjustment in COOT^58^ (version 0.9.8). Models were validated with MolProbity^59^. Further details are given in Supplementary Table 2. Schematics of ALECT2 Ia and Ib folds in Supplementary Fig. 3a were prepared using atom2svg. The energy maps in Supplementary Fig. 3b were prepared using the Amyloid Illustrator software^60^.

### Buried surface area calculations

Buried surface area values were measured by “measure buriedarea” in UCSF ChimeraX^61^ (version 1.7.1) with a default probe sphere radius of 1.4 Å. To adequately estimate the buried surface area, one rung of a protofilament was analyzed against eleven rungs of the opposing protofilament.

### Reporting summary

Further information on research design is available in the Nature Portfolio Reporting Summary linked to this article.

## Supplementary information

Supplementary Information

Reporting Summary

Transparent Peer Review file

## Source data

Source Data

## Data Availability

Cryo-EM maps have been deposited in the Electron Microscopy Data Bank (EMDB) under accession numbers EMD-66046 for ALECT2 type Ia, EMD-66047 for ALECT2 type Ib, EMD-66048 for ALECT2 type IIa, EMD-66049 for ALECT2 type IIb, and EMD-66050 for ALECT2 type III. Corresponding refined atomic models have been deposited in the Protein Data Bank (PDB) under accession numbers 9WL5 for ALECT2 type Ia, 9WL6 for ALECT2 type Ib, 9WL7 for ALECT2 type IIa, 9WL8 for ALECT2 type IIb, and 9WL9 for ALECT2 type III. The previously published structures used in this study are available in the Protein Data Bank under the following accession codes 5B0H (crystal structure of native LECT2) and 8G2V (in vitro–formed ALECT2 filaments). Mass spectrometry data have been deposited to the ProteomeXchange Consortium via the iProX partner repository under accession code PXD072679. The genotyping sequencing data for the rs31517 variant (I40V) have been deposited in GenBank under accession numbers PZ200919 for case 1, PZ200920 for case 2, PZ200921 for case 3, PZ200922 for case 4, and PZ200923 for case 5. The source data underlying Fig. 3d and Supplementary Figs. 1b, d, 2c, and 4c–e are provided as a Source Data file. Source data are provided with this paper.
