# Supplementary material for: Cryo-EM structures of ALECT2 filaments from human renal biopsies
Source: Nat Commun. 2026 May 22;17:6749. doi: 10.1038/s41467-026-73602-2 (PMC13385698; doi:10.1038/s41467-026-73602-2)
Supplement: Supplementary file 1 — Supplementary Information [file 41467_2026_73602_MOESM1_ESM.pdf]

Supplementary information

Cryo-EM Structures of ALECT2 Filaments from Human Renal Biopsies

Jian-Lin Zheng[1,2,3,†], Yu-Xin Zheng[1,2,3,†], Kai Chen[4,5†], Shuang Wang[4,5], Jia-Wei Liang[1,2,3], Su-Xia Wang[4,5], Li Yang[5,*], Yang Shi[1,2,3,*]

Affiliations:

[1] Department of Pathology of the First Affiliated Hospital and School of Brain Science and Brain Medicine, Zhejiang University School of Medicine, Hangzhou, 310058, China.

[2] Liangzhu Laboratory, MOE Frontier Science Center for Brain Science and Brain-machine Integration, State Key Laboratory of Brain-machine Intelligence, Zhejiang University, 1369 West Wenyi Road, Hangzhou 311121, China.

[3] NHC and CAMS Key Laboratory of Medical Neurobiology, Zhejiang University, Hangzhou 310058, China.

[4] Laboratory of Electron Microscopy, Pathological Center, Peking University First Hospital, 100034, Beijing, China.

[5] Renal Division, Department of Medicine, Peking University First Hospital; Renal Pathological Center, Institute of Nephrology, Peking University; Key Laboratory of Chronic Kidney Disease (CKD) Prevention and Treatment, Ministry of Education of China; Key Laboratory of Renal Diseases, Ministry of Health of China, 100034, Beijing, China.

† These authors contributed equally to this work.

* Correspondence: yshi22@zju.edu.cn (Yang Shi), li.yang@bjmu.edu.cn (Li Yang)

Table of contents:

Supplementary Figures 1-7

Supplementary Tables 1-4

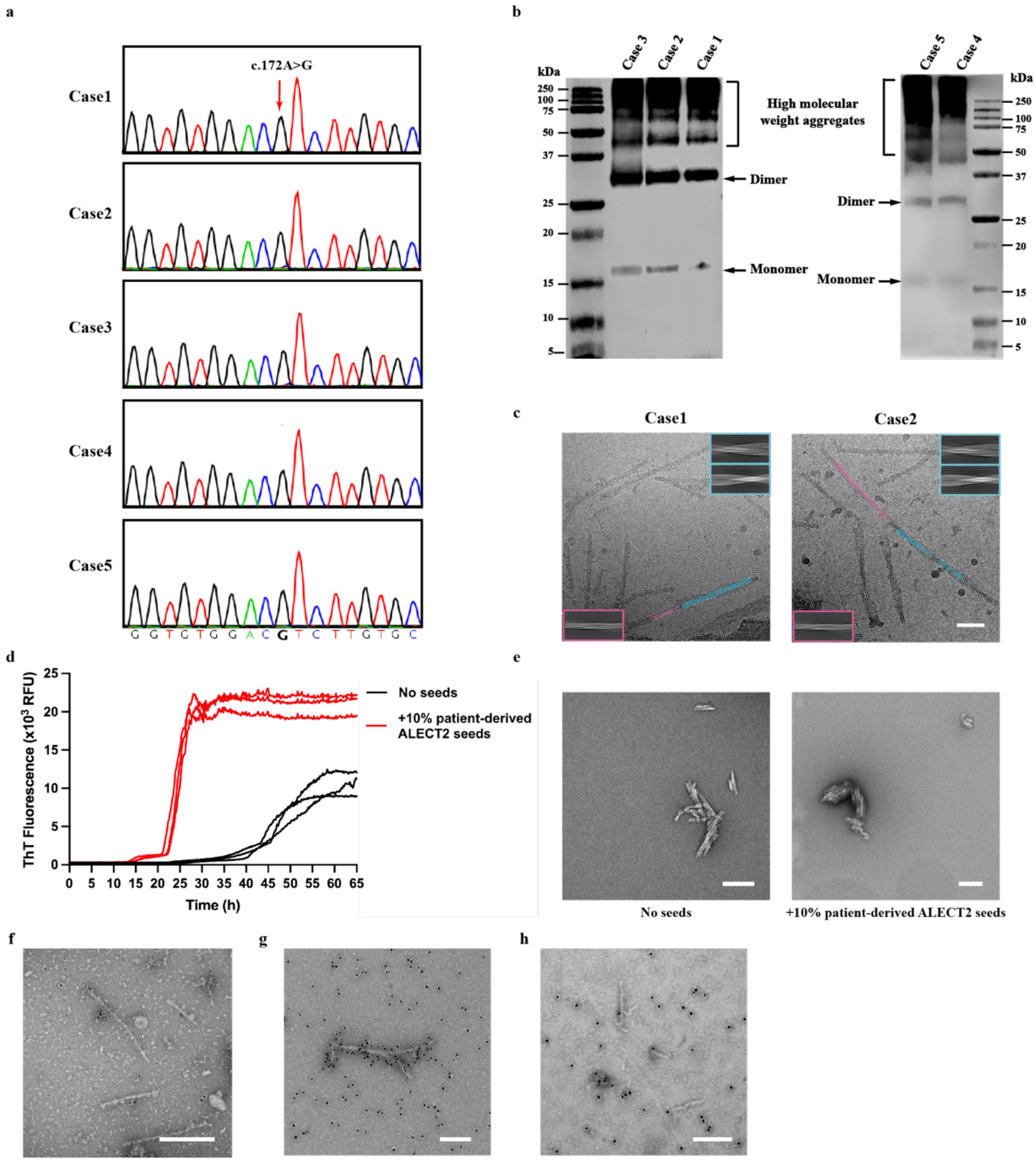

Supplementary Fig. 1 Genetic and biochemical characterizations. **a** DNA sequencing of exon 3 of *LECT2* gene from five cases, confirming homozygosity for the G allele at nucleotide 172 (c.172A>G). The missense SNP site is indicated by a red arrow. **b** Immunoblotting of water-extracts from renal biopsy samples of five cases, showing bands corresponding to LECT2 monomers (~16 kDa), dimers (~32 kDa) and higher-molecular-weight species. **c** Representative cryo-EM micrographs of ALECT2 filaments from cases 1 and 2. A 2D class average of unpaired filaments is shown at the bottom left of each panel, and that of paired filaments at the top right. Coexisting paired (cyan) and unpaired (hot pink) filaments are highlighted,

with the contributing segments traced. Scale bars, 50 nm. **d** Amyloid seeding assay of recombinant LECT2 monitored by ThT fluorescence in the absence (black) or presence (red) of 10% patient-derived ALECT2 seeds (w/w). All replicates are shown (n = 3). RFU, relative fluorescence units. **e** Electron micrographs of recombinant LECT2 after 65 h of incubation in the amyloid seeding assay, in the absence (left) or presence (right) of 10% patient-derived ALECT2 seeds. Scale bars, 200 nm. **f**-**h** Representative negative-stain immunogold labeling micrographs of patient-derived ALECT2 filaments (**f**), and *in vitro* assembled LECT2 fibrils before (**g**) and after (**h**) proteinase K digestion. Scale bar, 200 nm. Source data are provided as a Source Data file.

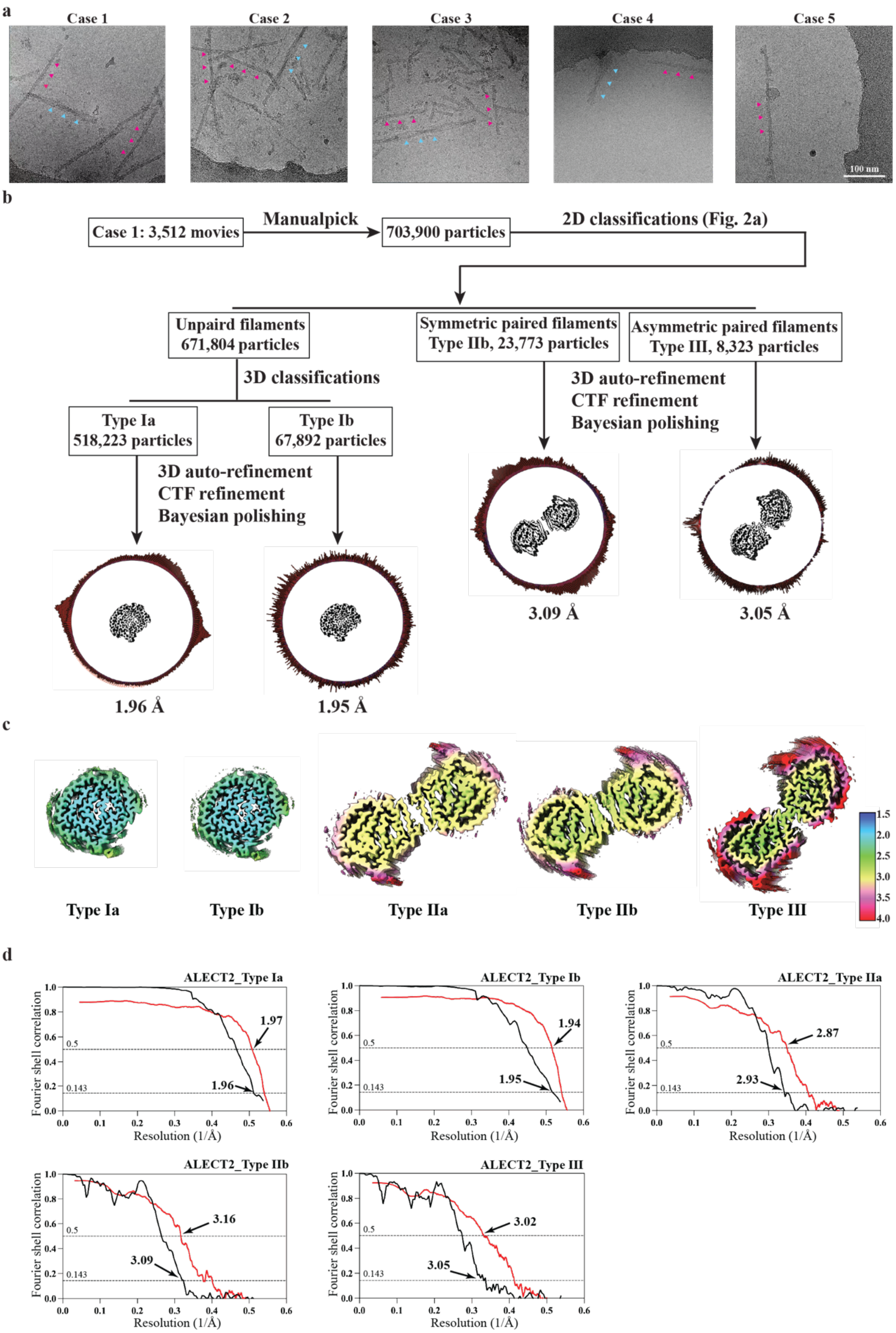

Supplementary Fig. 2 Cryo-EM data processing of ALECT2 filaments. **a** Representative cryo-EM micrographs of patient-derived ALECT2 filaments from cases 1–5. Unpaired and paired filaments are indicated by pink and

blue triangles, respectively. **b** Representative cryo-EM image-processing workflow for case 1, with the angular distribution of particles shown for the final reconstruction. **c** Local resolution maps of ALECT2 filaments. **d** Fourier shell correlation (FSC) curves for cryo-EM maps and atomic models of ALECT2 filaments. FSC curves for two independently refined cryo-EM half maps are shown in black, and for the final refined atomic model against the final cryo-EM map in red. Source data are provided as a Source Data file.

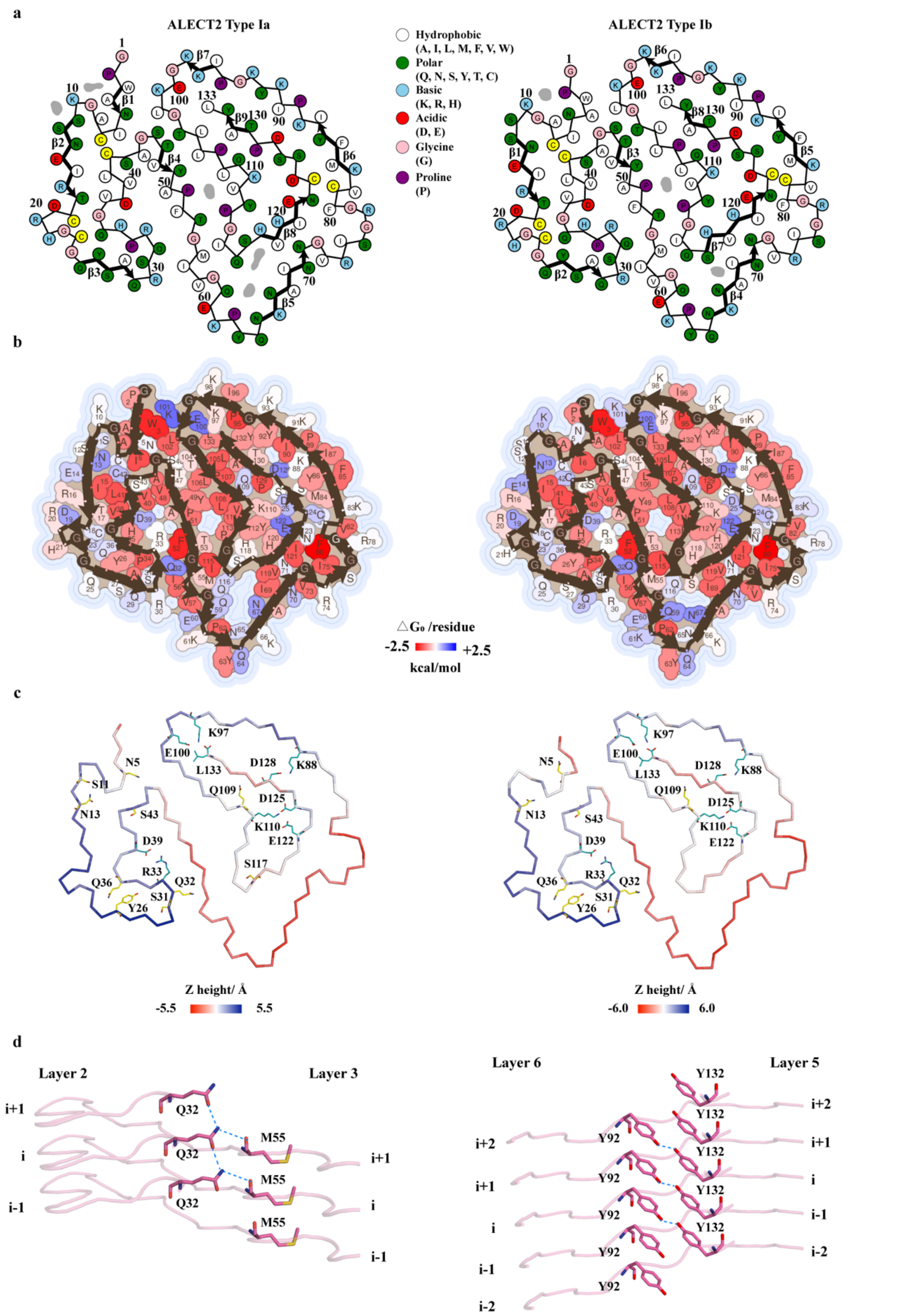

Supplementary Fig. 3 Schematic representations of ALECT2 type Ia and type Ib protofilaments.

a Schematics of ALECT2 Ia (left) and Ib (right) folds. Negatively charged

residues are in red, positively charged residues in blue, polar residues in green, non-polar residues in white, prolines in purple, and glycines in pink. Thick connecting lines with arrowheads indicate β-strands. **b** Schematic energy maps for Ia (left) and Ib (right) folds. **c** Structure of ALECT2 Ia (left) and Ib (right) filaments, with the main chain colored according to its height along the filament axis (red to blue, low to high). Residues involved in side-chain-main-chain interactions are shown in yellow, and buried charged residues are shown in teal. **d** Representative axial inter-molecular interactions within the ALECT2 filaments.

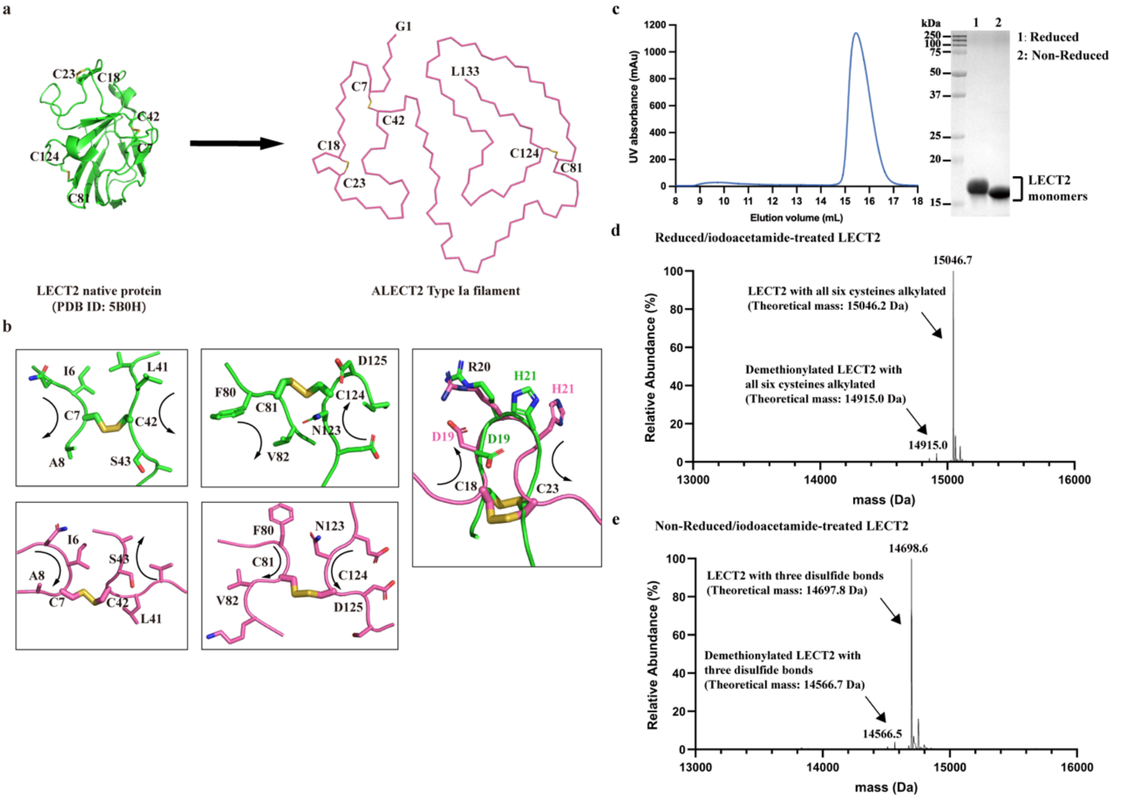

Supplementary Fig. 4 Disulfide bonds in LECT2.

a Structures of LECT2 in the native and fibrillar states. The crystal structure of native LECT2 (PDB ID: 5B0H) is shown in green, and the patient-derived ALECT2 filament structure (type Ia) is shown in hot pink. Three pairs of intramolecular disulfide bonds are labeled. **b** Comparison of the local structures surrounding the three pairs of intramolecular disulfide bonds in native and fibrillar LECT2, colored as in **a**. The N- to C-terminal directions are indicated by black arrows. **c** Size-exclusion chromatography profile (left) and SDS–PAGE analysis of refolded LECT2 under non-reducing and reducing conditions (right). **d**, **e** Mass spectra of undigested refolded LECT2-I40V under reducing (**d**) and non-reducing (**e**) conditions. Source data are provided as a Source Data file.

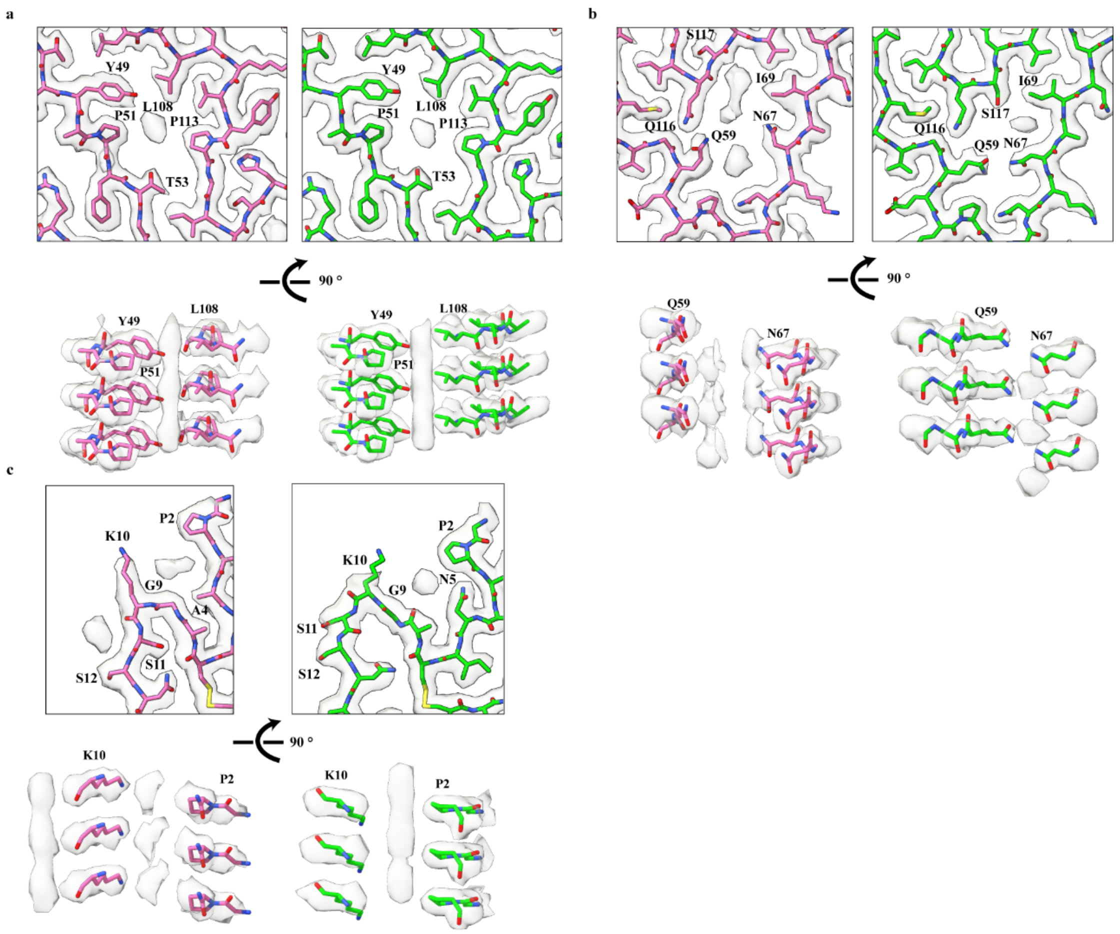

Supplementary Fig. 5 Additional densities in ALECT2 filaments.

a-c Top views (upper) and side views (lower) of additional densities within two cavities formed by residues Y49, P51, T53, P113, and L108 (**a**); Q116, Q59, N67, and I69 (**b**); and two grooves formed by residues P2, A4 (type Ia), N5 (type Ib), G9, and K10; K10 and S12 (**c**). The Ia fold is shown in hot pink and the Ib fold in lime.

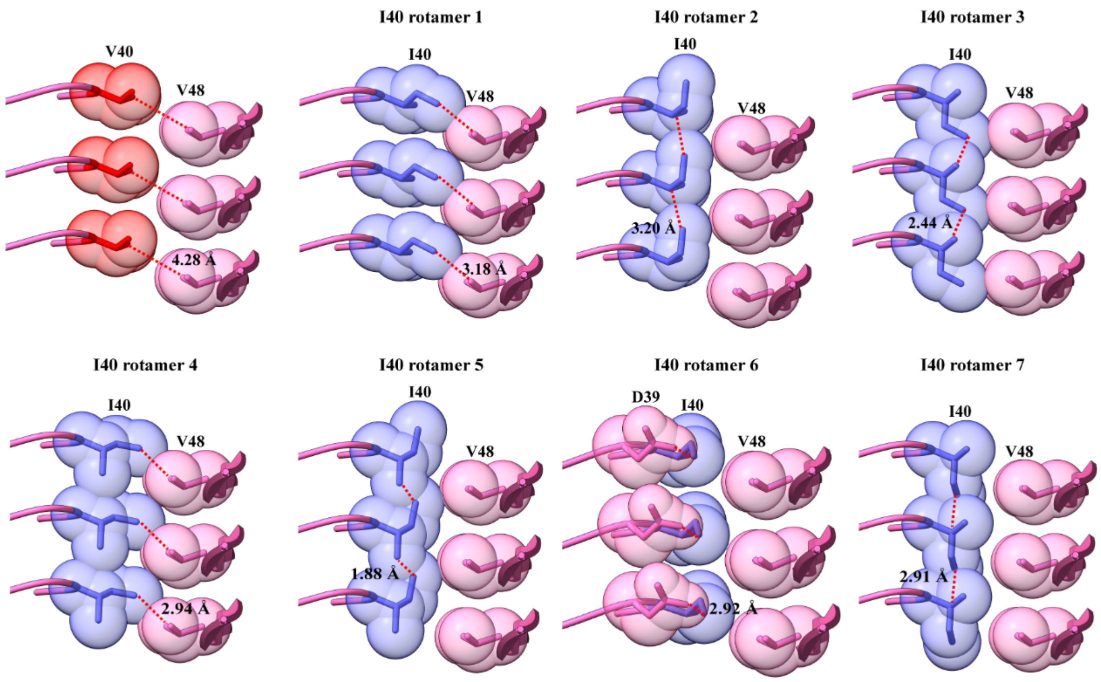

Supplementary Fig. 6 Structural incompatibility of LECT2-I40 with filaments formed by LECT2-V40.

Close-up views of residue 40 in ALECT2 filament models. Residue 40 is modeled with valine (V40, top-left panel, red) and isoleucine (I40, blue, seven rotamers ranked by probability). Neighboring residues are shown in pink. Side-chains are displayed as both sticks and spheres. Red dashed lines indicate the closest atomic contacts between the carbon atoms of residue 40 and neighboring residues.

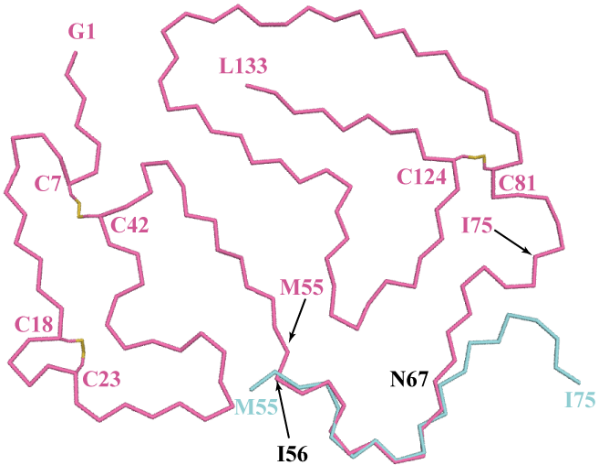

Supplementary Fig. 7 Comparison with recombinant ALECT2 filaments.

Structural comparison of the patient-derived ALECT2 Ia fold (hot pink) and *in vitro* formed ALECT2 filaments (PDB 8G2V, cyan). Alignments were performed over residues I56-N67.

Supplementary Table 1 | Demographic and clinical features of five patients with ALECT2 amyloidosis.

Pt NO.	Age (years)/Sex	BMI (kg/m^2)	Ethnic origin	Hypert-ension	DM	Scr (μmol/L)	eGFR (mL/min /$1.73m^2$)	Alb (g/L)	UTP (g/24h)	Microscopic hematuria
1	73/M	24.54	Han	+	+	179.00	31.88	31.10	3.00	-
2	65/M	26.18	Han	+	-	191.50	30.88	39.00	NA	-
3	58/M	29.63	Han	+	-	230.00	26.00	39.50	1.82	-
4	65/M	23.81	Han	-	-	146.44	42.72	40.80	0.41	-
5	79/M	19.82	Han	+	-	541.80	7.96	24.70	7.92	+

Pt: patient; M: male; BMI: body mass index; DM: diabetes mellitus; Scr: serum creatinine; eGFR: estimated glomerular filtration rate; Alb: serum albumin; UTP: urine total protein; -: negative; +: positive; NA: not available. Reference ranges for healthy adults are as follows: Scr, 44-133 μmol/L; eGFR, ≥90 $mL \cdot min^{-1} \cdot 1.73\ m^{-2}$; Alb, 40-55 g/L; UTP, 0-0.15 g/24 h.

Supplementary Table 2 | Cryo-EM data collection, refinement and validation.

	ALECT2 Type Ia (Case 1)	ALECT2 Type Ib (Case 1)	ALECT2 Type IIa (Case 2)	ALECT2 Type IIb (Case 1)	ALECT2 Type III (Case 1)
**Data collection and processing**					
Electron Gun	CFEG	CFEG	CFEG	CFEG	CFEG
Detector	Falcon 4i	Falcon 4i	Falcon 4i	Falcon 4i	Falcon 4i
Energy filter slit width (eV)	10	10	10	10	10
Magnification	130 kx	130 kx	130 kx	130 kx	130 kx
Voltage (kV)	300	300	300	300	300
Electron exposure (e–/Å^2)	42	42	42	42	42
Defocus range (μm)	1.2-1.6	1.2-1.6	1.2-1.6	1.2-1.6	1.2-1.6
Pixel size (Å)	0.93	0.93	0.93	0.93	0.93
Initial particle images (no.)	703,900	703,900	505,253	703,900	703,900
Symmetry imposed	C1	C1	C2	C1	C1
Final particle images (no.)	518,223	67,892	24,647	23,773	8,323
Map resolution (Å)/ FSC: 0.143	1.96	1.95	2.93	3.09	3.05
Helical rise (Å)	4.81	4.82	4.77	2.38	4.78
Helical twist (°)	-1.02	-0.94	-0.91	179.524	-0.90
**Refinement**					
Initial model used (PDB code)	-	-	-	-	-
Model resolution (Å)/FSC: 0.5	1.97	1.94	2.87	3.16	3.02
Map sharpening *B* factor (Å^2)	-35.26	-23.17	-47.79	-47.37	-36.12
**Model composition**					
Non-hydrogen atoms	3,066	3,066	6,132	6,132	6,132
Protein residues	399	399	798	798	798
Ligands	0	0	0	0	0
Protein *B* factors (Å^2)	29.34	31.22	68.64	54.87	49.15
R.m.s. deviations					
Bond lengths (Å)	0.004	0.005	0.005	0.003	0.004
Bond angles (°)	0.749	1.002	1.054	0.822	0.793
Validation					
MolProbity score	1.99	1.77	1.91	1.99	2.00
Clash score	9.16	5.56	6.55	7.77	9.98
Poor rotamers (%)	0	0	0	0	0
Ramachandran plot					
Favored (%)	91.60	92.37	90.08	89.31	92.24
Allowed (%)	8.40	7.63	9.92	10.69	7.76
Disallowed (%)	0	0	0	0	0
PDB	9WL5	9WL6	9WL7	9WL8	9WL9
EMDB	66046	66047	66048	66049	66050

Supplementary Table 3 | Structural alignment details.

Models aligned	Residues aligned	# atom pairs	r.m.s.d.(Å)
ALECT2 type Ia & ALECT2 type Ib	1-133	532	1.027
ALECT2 type Ia & ALECT2 type IIa	1-133	532	0.249
ALECT2 type Ia & ALECT2 type IIb	1-133	532	0.283
ALECT2 type Ia & ALECT2 type III-pf-A	1-133	532	0.186
ALECT2 type Ia & ALECT2 type III-pf-B	1-133	532	0.156
ALECT2 type Ia & 8G2V	55-75	84	3.834
ALECT2 type Ia & 8G2V	56-67	48	0.762

Supplementary Table 4 | Disulfide-linked peptides identified in recombinant LECT2-I40V by LC–MS/MS.

Disulfide pairs	Disulfide-linked peptides	Experimental mass (Da)	Theoretical mass (Da)
Cys7-Cys42	(-1-10) - (31-74)	5875.8709	5875.8673
	(-1-10) - (31-66)	5037.4211	5037.4264
	(1-10) - (31-66)	4906.3876	4906.3860
	(-1-16) - (31-74)	6562.1958	6562.2021
	(-1-16) - (31-66)	5723.7647	5723.7612
	(1-16) - (34-66)	5221.4869	5221.529
	(1-16) - (34-74)	6059.9334	6059.9699
Cys81-Cys124	(75-83) - (102-133)	4472.2758	4472.2739
	(79-91) - (111-133)	4116.0046	4116.0066
	(75-83) - (111-133)	3508.6643	3508.6622
	(75-91) - (102-133)	5492.8528	5492.8569
	(75-91) - (111-133)	4529.2464	4529.2452
	(75-83) - (99-133)	4786.481	4786.4329
	(75-83) - (98-133)	4914.5647	4914.5279
Cys18-Cys23	(11-20) - (21-30)	2283.9954	2283.9941
	(1-20) - (21-30)	3281.491	3281.4744
	(17-30)	1579.6495	1579.6488
	(11-30)	2265.9852	2265.9835
	(1-30)	3263.4801	3263.4638
Cys7-Cys23	(-1-10) - (21-30)	2250.9961	2250.9953
	(1-20) - (21-30)	3281.4948	3281.4744
	(1-16) - (17-30)	3281.4831	3281.4744
Cys23-Cys23	(21-30) - (21-30)	2209.9319	2209.9362
Cys23-Cys124	(21-30) - (111-133)	3648.6236	3648.6229
Cys18-Cys42	(11-20) - (31-66)	5070.423	5070.4253
Cys7-Cys7	(1-10) - (1-10)	2029.9831	2029.9734
Cys42-Cys81	(31-61) - (75-88)	4908.4371	4908.4454
Cys18-Cys124	(11-20) - (98-133)	5128.5366	5128.5464
