# Supplementary material for: Cryo-EM structures of ALECT2 filaments from human renal biopsies
Source: Nat Commun. 2026 May 22;17:6749. doi: 10.1038/s41467-026-73602-2 (PMC13385698; doi:10.1038/s41467-026-73602-2)
Supplement: Supplementary file 2 — Reporting Summary [file 41467_2026_73602_MOESM2_ESM.pdf]

Corresponding author(s): Yang Shi, Li Yang

Last updated by author(s): Mar 30, 2026

Reporting Summary

Nature Portfolio wishes to improve the reproducibility of the work that we publish. This form provides structure for consistency and transparency in reporting. For further information on Nature Portfolio policies, see our Editorial Policies and the Editorial Policy Checklist.

Statistics

For all statistical analyses, confirm that the following items are present in the figure legend, table legend, main text, or Methods section.

n/a	Confirmed	
☐	☒	The exact sample size (*n*) for each experimental group/condition, given as a discrete number and unit of measurement
☐	☒	A statement on whether measurements were taken from distinct samples or whether the same sample was measured repeatedly
☒	☐	The statistical test(s) used AND whether they are one- or two-sided<br>*Only common tests should be described solely by name; describe more complex techniques in the Methods section.*
☒	☐	A description of all covariates tested
☒	☐	A description of any assumptions or corrections, such as tests of normality and adjustment for multiple comparisons
☐	☒	A full description of the statistical parameters including central tendency (e.g. means) or other basic estimates (e.g. regression coefficient) AND variation (e.g. standard deviation) or associated estimates of uncertainty (e.g. confidence intervals)
☒	☐	For null hypothesis testing, the test statistic (e.g. *F*, *t*, *r*) with confidence intervals, effect sizes, degrees of freedom and *P* value noted<br>*Give P values as exact values whenever suitable.*
☒	☐	For Bayesian analysis, information on the choice of priors and Markov chain Monte Carlo settings
☒	☐	For hierarchical and complex designs, identification of the appropriate level for tests and full reporting of outcomes
☒	☐	Estimates of effect sizes (e.g. Cohen's *d*, Pearson's *r*), indicating how they were calculated

Our web collection on statistics for biologists contains articles on many of the points above.

Software and code

Policy information about availability of computer code

Data collection	EPU (v3.8)
Data analysis	RELION (v5.0), CTFFIND (v4.1), COOT (v0.9.8), PHENIX (v1.21-5207-000), Pymol (v3.1.1), ChimeraX (v1.7.1), atom2svg (https://doi.org/10.5281/zenodo.4090924), Amyloid Illustrator software (https://zenodo.org/records/15218932).

For manuscripts utilizing custom algorithms or software that are central to the research but not yet described in published literature, software must be made available to editors and reviewers. We strongly encourage code deposition in a community repository (e.g. GitHub). See the Nature Portfolio guidelines for submitting code & software for further information.

Data

Policy information about availability of data

All manuscripts must include a data availability statement. This statement should provide the following information, where applicable:

- Accession codes, unique identifiers, or web links for publicly available datasets
- A description of any restrictions on data availability
- For clinical datasets or third party data, please ensure that the statement adheres to our policy

Cryo-EM maps have been deposited in the Electron Microscopy Data Bank (EMDB) under accession numbers EMD-66046 for ALECT2 type Ia, EMD-66047 for ALECT2 type Ib, EMD-66048 for ALECT2 type IIa, EMD-66049 for ALECT2 type IIb, EMD-66050 for ALECT2 type III. Corresponding refined atomic models have been deposited in the Protein Data Bank (PDB) under accession numbers 9WL5 for ALECT2 type Ia, 9WL6 for ALECT2 type Ib, 9WL7 for ALECT2 type IIa, 9WL8 for ALECT2

type IIb, 9WL9 for ALECT2 type III. The mass spectrometry proteomics data have been deposited to the ProteomeXchange Consortium (https://proteomecentral.proteomexchange.org) via the iProX partner repository with the dataset identifier PXD072679. The genotyping sequencing data for the rs31517 variant (I40V) have been deposited in GenBank under accession number PZ200919 for case 1, PZ200920 for case 2, PZ200921 for case 3, PZ200922 for case 4, and PZ200923 for case 5.

Research involving human participants, their data, or biological material

Policy information about studies with human participants or human data. See also policy information about sex, gender (identity/presentation), and sexual orientation and race, ethnicity and racism.

Reporting on sex and gender	See Supplementary Table 1. The patients were all male.
Reporting on race, ethnicity, or other socially relevant groupings	All participants in this study were of Chinese descent.
Population characteristics	See Supplementary Table 1.
Recruitment	See the Methods section. Samples were obtained from patients with ALECT2 amyloidosis according to tissue availability.
Ethics oversight	The use of samples in this study was approved by the ethics committee at the Peking University First Hospital (approval number 2022[448-002]). Informed consent was obtained from all patients.

Note that full information on the approval of the study protocol must also be provided in the manuscript.

Field-specific reporting

Please select the one below that is the best fit for your research. If you are not sure, read the appropriate sections before making your selection.

☒ Life sciences ☐ Behavioural & social sciences ☐ Ecological, evolutionary & environmental sciences

For a reference copy of the document with all sections, see nature.com/documents/nr-reporting-summary-flat.pdf

Life sciences study design

All studies must disclose on these points even when the disclosure is negative.

Sample size	Renal tissue samples were obtained from patients with ALECT2 amyloidosis during biopsy (n=5) . Samples were chosen based on tissue availability.
Data exclusions	Pre-established common image classification procedures (S.H.W. Scheres, J. Struc. Biol. 180: 519-530, (2012)) were employed to select particle images with the highest resolution content in the cryo-EM reconstruction process. Details of the number of selected images are given in table S2.
Replication	For ALECT2 amyloidosis, we examined five biological replicates, and representative data are shown as described in the main text.
Randomization	As this study was not designed to compare different treatment groups, randomization was not applicable.
Blinding	The investigators were not blinded to allocation during experiments and outcome assessment. The perceived risk of detection/performance bias was deemed negligible.

Reporting for specific materials, systems and methods

We require information from authors about some types of materials, experimental systems and methods used in many studies. Here, indicate whether each material, system or method listed is relevant to your study. If you are not sure if a list item applies to your research, read the appropriate section before selecting a response.

Materials & experimental systems

n/a	Involved in the study
☐	☒ Antibodies
☒	☐ Eukaryotic cell lines
☒	☐ Palaeontology and archaeology
☒	☐ Animals and other organisms
☒	☐ Clinical data
☒	☐ Dual use research of concern
☒	☐ Plants

Methods

n/a	Involved in the study
☒	☐ ChIP-seq
☒	☐ Flow cytometry
☒	☐ MRI-based neuroimaging

Antibodies

Antibodies used

See the Methods section. For immunohistochemistry and tissue-section immunogold labeling, an anti-human LECT2 polyclonal antibody (AF722, R&D Systems) was used at a 1:40 dilution. For immunoblots, an anti-human LECT2 polyclonal antibody (AF722, R&D Systems) was used at a 1:1,000 dilution.

Validation

The anti-human LECT2 polyclonal antibody (AF722, R&D Systems) was validated against aggregated LECT2 in manufacturer's datasheet (R&D Systems).

Plants

Seed stocks

Not relevant to study.

Novel plant genotypes

Not relevant to study.

Authentication

Not relevant to study.
