# Supplementary material for: Cryo-EM structures of ALECT2 filaments from human renal biopsies
Source: Nat Commun. 2026 May 22;17:6749. doi: 10.1038/s41467-026-73602-2 (PMC13385698; doi:10.1038/s41467-026-73602-2)
Supplement: Supplementary file 3 — Transparent Peer Review file [file 41467_2026_73602_MOESM3_ESM.pdf]

nature portfolio

Peer Review File

Cryo-EM Structures of ALECT2 Filaments from Human Renal Biopsies

Corresponding Author: Dr Yang Shi

This file contains all reviewer reports in order by version, followed by all author rebuttals in order by version.

Version 0:

Reviewer comments:

Reviewer #1

(Remarks to the Author)
In NCOMMS-25-77218, Zheng et al. extracted LECT2 filaments from kidney biopsies of five ALECT2 patients and determined 16 filament structures at 1.9 Angstrom to 3.4 Angstrom resolution. They observed at least five protofilament types: two monomeric types (Ia and Ib) and three dimeric types (IIa, IIb, and III) Type Ia was the most prevalent across all five biopsy samples, with the other types making up a minor population of fibrils.

The structural data are of high quality, and it is exciting that all amino acids were visible in the ex vivo fibrils (in contrast to a previously published structure of fibrils prepared in vitro). This work should be of high interest to readers of Nature Communications. I recommend publication once the authors have considered the following points.

The authors may wish to comment on a preprint by the Saelices group in which they report the cryoEM structures of LECT2 amyloid fibrils extracted from human kidney (https://doi.org/10.1101/2025.07.15.664973). Overall, the structures appear to be similar, but the Saelices paper observed that the single- and double-protofilament morphology co-existed within a single fibril, and that there was acetylation of some Lys side chains. The authors should note whether they also observed the former, or if single- and double-protofilaments were separate molecules.

As written, the results regarding the in vitro prepared fibrils don't add much to the paper, other than to show the low-resolution structure of in vitro refolded aggregates is much different than that of the ex vivo material (which was already evident from ref #11). It would be more informative if the authors were to characterize the state of disulfide bond formation. They reduced the disulfides then attempted to reoxidize them, but to what extent was this successful? Did they reform at all, and if so, were they scrambled or natively paired? Since the current work and that of the Saelices group have established that the disulfides remain intact and correctly paired in the physiological fibrils, the relevance of structures in which they are reduced/scrambled is questionable.

The discussion of 'fuzzy coats' and their relationship with antibody binding should be clarified and perhaps toned down. The general readership may not be familiar with this terminology so it should be defined. The authors seem to equate the presence of a fuzzy coat with antibody binding, and the lack of a fuzzy coat with lack of antibody binding (e.g., lines 48-151). In the present case, poor antibody binding is likely explained by the near complete conversion of native structure to non-native structure, not the absence of a fuzzy coat per se.

From the discussion regarding carboxyl group protonation, low pH conditions of in vitro aggregation, and lines 279-298, the reader may be left with the impression that fibril formation of LECT2 is driven by strongly acidic pH that is only present within the lysosome. To my knowledge, there is no evidence that LECT2 is trafficked to lysozomes. In fact, mildly acidic conditions weaken zinc binding via histidine protonation and promote fibrillization as pointed out by the authors and ref #21. The authors should clarify the relationship of pH, histidine vs. carboxyl group protonation, the distinct mechanisms by which those events may lead to aggregation, and the extent to which those conditions may be present in the body.

How are the six layers defined (e.g., Fig. 3B)? On a related point, the authors describe the interactions that stabilize the fold within one protomer, and between dimers in type II structures, in detail, but there is relatively little treatment of the interactions that stabilize successive protomers along the fibrillar axis. Expanding the latter discussion would be welcome.

Minor comments: In Fig. 3B, K30 should be R30. In Supplementary Table 1, it would be useful to provide the normal range of the clinical values.

Reviewer #2

(Remarks to the Author)
The article by Zheng et al. describing the structural determination of ALECT2 fibrils from human kidney disease biopsies is timely and of high interest to the systemic amyloid diseases community. As a recently discovered amyloidosis, ALECT2 is critically understudied and under diagnosed. This work is further unique in its ability to discern structures of ALECT2 fibrils from living patient biopsies.

The article is clear and compelling, and, in my opinion, would be suitable for publication provided the following questions were addressed.

The suggestion that a common ALECT2 fibril fold is due to a common cause for their amyloid structure is important, since unambiguous diagnosis would now presumably be accessible via structure determination from biopsies. It would be useful for the authors to further expand on the conditions that would allow this unique characterization of fibrils from live patient biopsies.

The authors note that two clusters of residues bury charges in the core of the ALECT2 fibrils. How is the protonation state of the residues determined in such a case? In other words, is it certain that the aspartate or glutamate residues in the clusters are not protonated?

The authors further suggest that the ALECT2 fibrils may have formed under acidic conditions, implying that the local pKa of some residues is altered int he fibril core. If so, we would expect that altering the pH of the solution containing the fibrils might destabilize them. Is this the case?

The structures determined are of exceptional quality, indicating the identity and geometry of all core residues, and revealing unknown bound densities. While some of those could easily be explained as bound ions or waters, are the authors certain an alternate backbone configuration would not satisfy the oblong density near S11 in their 1a fibril?

How do the authors rule out that the paired 1a fibrils are adventitious or that their proportion with respect to unpaired counterparts is a result of purification?

The authors indicate several reasons why it is advantageous to determine structures of patient-derived LECT2 fibrils, noting that in vitro generated fibrils lack the complexity of the former. Can the authors make a similar claim about in vitro grown recombinant LECT2 fibrils seeded with those extracted from biopsies? In other words, are the biopsy-extracted fibrils seed competent?

It would be helpful to have some details about the interfaces found in the paired ALECT2 fibrils, for example the shape complementarity and buried surface area per layer, compared to other such interfaces in patient derived amyloid fibrils.

In Supplementary table 2, it would be helpful to note what structures are determined from the same dataset.

Reviewer #3

(Remarks to the Author)
The manuscript reports cryo-EM structures of patient-derived ALECT2 amyloid filaments isolated from patient renal biopsies, describing six-layered protofilament folds with conservation of disulfide bonds. The authors also document minor polymorphisms and dimeric assemblies. Understanding the structures of amyloid fibrils formed by LECT2 is important for establishing disease mechanism, improving diagnostics and ultimately increasing the chance of improved clinical outcomes in the long term. The finding of a shared fold across unrelated cases is interesting and can direct targeting efforts. The authors also report inability to reproduce the patient fribrils with recombinant LECT2 - that is an important finding since in vitro fibrils are usually the material of choice for drug discovery workflows. In my opinion the article merits publication but I have a few comments to be addressed.

The current view of the FSC as reported in Supplementary figure 2 does not allow the reader a proper assessment of overfitting. For the Type Ia and Type Ib, the FSC for the maps do not seem to properly reach zero and just come to a sudden stop. Please report more complete curves for all reconstructions.

The conservation of the disulfides is quite curious. It would be nice to have a figure highlighting those in a representative

fibril and showing the structure of native LECT2 fold and its disulfide bridges for comparison.

The manuscript argues that commonly used anti-LECT2 antibodies (presumably raised against the native fold) give inconsistent immunohistochemical detection results because fibrillar ALECT2 incorporates all residues into the ordered cor and that reducies epitope accessibility. Building on your structural insights from this article, could the authors assess or at least speculate whether currently available monoclonal antibodies (that recognize distinct epitopes) could perform better for the detection of the amyloids described in the paper?

Minor point:

40The absence of a fuzzy coat
41 reduces antibody binding to ALECT2 filaments, which may explain the
42 inconsistent immunohistochemical detection and highlight the need for
43 conformation-specific antibodies for accurate diagnosis.f a fuzzy coat
41 reduces antibody binding to ALECT2 filaments, which may explain the
42 inconsistent immunohistochemical detection and highlight the need for
43 conformation-specific antibodies for accurate diagnosis.

I find this sentence confusing. I suggest something along the lines of: “Because all residues are incorporated into the fibril core, epitope accessibility is limited. This can explain variability in immunohistochemical detection and highlight the need for conformation-specific antibodies for improving accuracy in diagnosis”

Version 1:

Reviewer comments:

Reviewer #1

(Remarks to the Author)
The new experiments to characterize the state of disulfide bond formation of the in vitro prepared fibrils are a welcome addition. The data suggest a predominant population of native pairings with some scrambled disulfides present. This finding reinforces the importance of in vivo conditions to LECT2 amyloid structure.

The discussion of the protonation states of buried ionizable groups has been expanded, although I find it still to be speculative and a bit unclear. It would be *less* energetically favorable if D39 and D128 were protonated in their respective salt bridges, as this would leave buried, unsatisfied positive charges from the Lys. Are the authors suggesting that these carboxylates are protonated in the fibrils? For the K110-E122-D125 and K97-E100-C-term charge clusters, protonating both carboxylates in each cluster would be energetically costly for the same reason, so are the authors speculating that only a single carboxylate is protonated in each cluster? There is a lot of hand waving going on without any direct evidence. The authors do state this on lines 391-392, however, so overall this section is probably fine albeit speculative.

The authors addressed the remainder of my questions/concerns well. I recommend this paper for publication in Nature Communications.

Reviewer #2

(Remarks to the Author)
The authors have suitably addressed my comments.

Their revisions raise only one other minor question, regarding the identity of their bound densities: Would a waters placed at those positions be within hydrogen bonding distance of nearby residues? Particularly for the density near S11?

Reviewer #3

(Remarks to the Author)
In my opinion, the authors have addressed the major points of concerns raise by me and the other reviewers. I am happy to recommend the manuscript for publication

Version 2:

Reviewer comments:

Reviewer #1

(Remarks to the Author)
The authors have responded to my concerns adequately.

Reviewer #2

(Remarks to the Author)
I find the revisions suitable and appreciate the authors' efforts to improve their manuscript.

Open Access This Peer Review File is licensed under a Creative Commons Attribution 4.0 International License, which permits use, sharing, adaptation, distribution and reproduction in any medium or format, as long as you give appropriate credit to the original author(s) and the source, provide a link to the Creative Commons license, and indicate if changes were made.
In cases where reviewers are anonymous, credit should be given to 'Anonymous Referee' and the source.
The images or other third party material in this Peer Review File are included in the article's Creative Commons license, unless indicated otherwise in a credit line to the material. If material is not included in the article's Creative Commons license and your intended use is not permitted by statutory regulation or exceeds the permitted use, you will need to obtain permission directly from the copyright holder.
To view a copy of this license, visit https://creativecommons.org/licenses/by/4.0/

We thank the reviewers for their constructive and helpful comments, which we feel have contributed to an improved manuscript.

Reviewer #1 (Remarks to the Author):

In NCOMMS-25-77218, Zheng et al. extracted LECT2 filaments from kidney biopsies of five ALECT2 patients and determined 16 filament structures at 1.9 Angstrom to 3.4 Angstrom resolution. They observed at least five protofilament types: two monomeric types (Ia and Ib) and three dimeric types (IIa, IIb, and III) Type Ia was the most prevalent across all five biopsy samples, with the other types making up a minor population of fibrils.

The structural data are of high quality, and it is exciting that all amino acids were visible in the ex vivo fibrils (in contrast to a previously published structure of fibrils prepared in vitro). This work should be of high interest to readers of Nature Communications. I recommend publication once the authors have considered the following points.

The authors may wish to comment on a preprint by the Saelices group in which they report the cryoEM structures of LECT2 amyloid fibrils extracted from human kidney (https://doi.org/10.1101/2025.07.15.664973). Overall, the structures appear to be similar, but the Saelices paper observed that the single- and double-protofilament morphology co-existed within a single fibril, and that there was acetylation of some Lys side chains. The authors should note whether they also observed the former, or if single- and double-protofilaments were separate molecules.

Afrin *et al.* recently reported, in a preprint, cryo-EM structures of *ex vivo* ALECT2 filaments extracted from a nephrectomy specimen of a patient with end-stage renal failure due to amyloid deposition. We now cite their paper in the first paragraph of the Discussion (lines 340-347; line numbers refer to the revised manuscript with accepted and highlighted changes).

The co-existing single- and double-protofilament morphologies in one filament can also be observed in our datasets, and we now show two representative micrographs from cases 1 and 2 in the revised manuscript (lines 137-139, Supplementary Fig. 1c).

As written, the results regarding the in vitro prepared fibrils don't add much to the paper, other than to show the low-resolution structure of in vitro refolded aggregates is much different than that of the ex vivo material (which was already evident from ref #11). It would be more informative if the authors were to characterize the state of disulfide bond formation. They reduced the disulfides then attempted to reoxidize them, but to what extent

was this successful? Did they reform at all, and if so, were they scrambled or natively paired? Since the current work and that of the Saelices group have established that the disulfides remain intact and correctly paired in the physiological fibrils, the relevance of structures in which they are reduced/scrambled is questionable.

SDS–PAGE and mass spectrometry were used to characterize the disulfide-bonding state of refolded LECT2. The corresponding data and descriptions have now been included in the revised manuscript (lines 238-248; Supplementary Fig. 4; Supplementary Table 4). In brief, both SDS-PAGE and LC-MS analysis of undigested refolded LECT2 showed that the predominant form contains three intramolecular disulfide bonds. LC-MS/MS analysis of trypsin-digested refolded LECT2 detected both native and non-native disulfide bond pairings, with their relative abundances not reliably quantifiable.

The discussion of 'fuzzy coats' and their relationship with antibody binding should be clarified and perhaps toned down. The general readership may not be familiar with this terminology so it should be defined. The authors seem to equate the presence of a fuzzy coat with antibody binding, and the lack of a fuzzy coat with lack of antibody binding (e.g., lines 48-151). In the present case, poor antibody binding is likely explained by the near complete conversion of native structure to non-native structure, not the absence of a fuzzy coat per se.

"Fuzzy coat" is now defined in the Introduction of the revised manuscript (lines 96-99). The immuno-EM results have been reorganized. Data related to patient-derived filaments are retained in the original paragraph (lines 162-168), whereas results for *in vitro*–formed filaments have been moved to a later section (lines 250-258). For the patient-derived filaments, the anti-LECT2 antibody (AF722) used in this study showed binding properties similar to those reported for tau-core antibodies: it weakly labels filaments in negative-stain immuno-EM but labels deposits in immunohistochemistry. This behavior supports the conclusion that the epitopes recognized by the antibodies used in this study are located within the filament core. The immuno-EM results for *in vitro*–formed filaments show binding properties distinct from those of patient-derived filaments, consistent with their structural differences. We also clarified the relationship between the presence of a fuzzy coat and antibody binding in the Discussion, with a more cautious interpretation (lines 417-430).

From the discussion regarding carboxyl group protonation, low pH conditions of in vitro aggregation, and lines 279-298, the reader may be left with the impression that fibril formation of LECT2 is driven by strongly

acidic pH that is only present within the lysosome. To my knowledge, there is no evidence that LECT2 is trafficked to lysozomes. In fact, mildly acidic conditions weaken zinc binding via histidine protonation and promote fibrillization as pointed out by the authors and ref #21. The authors should clarify the relationship of pH, histidine vs. carboxyl group protonation, the distinct mechanisms by which those events may lead to aggregation, and the extent to which those conditions may be present in the body.

We have rewritten the discussion on the role of pH (lines 374–395) to clarify that acidic pH may facilitate ALECT2 aggregation via two mechanisms: destabilization of native LECT2 via histidine protonation, and stabilization of ALECT2 filaments through carboxyl protonation.

How are the six layers defined (e.g., Fig. 3B)?
Most chains are arranged parallel to the direction of the main-chain between residues S46 and K61, which was therefore used as the axis to define structural layers for efficient description of the filament architecture. The layers were numbered starting from the N-terminal toward the opposite side, according to their positions in the filament structure rather than their order in the primary sequence (left to right in Fig. 3b). Similar layer-based descriptions have previously been applied to tau filaments (PMID: 34588692) and TMEM106B filaments (PMID: 35344985). The six-layer organization was already described in lines 174–178 of the original version, and a more detailed description has now been added to the legend of Fig. 3b.

On a related point, the authors describe the interactions that stabilize the fold within one protomer, and between dimers in type II structures, in detail, but there is relatively little treatment of the interactions that stabilize successive protomers along the fibrillar axis. Expanding the latter discussion would be welcome.

A new paragraph has been added to the Discussion (lines 349–360) to address the structural basis of axial stability in the filaments.

Minor comments: In Fig. 3B, K30 should be R30.

We have corrected K30 to R30 in Fig. 3B.

In Supplementary Table 1, it would be useful to provide the normal range of the clinical values.
The normal ranges are now provided in the footnotes of Supplementary Table 1.

Reviewer #2 (Remarks to the Author):

The article by Zheng et al. describing the structural determination of ALECT2 fibrils from human kidney disease biopsies is timely and of high interest to the systemic amyloid diseases community. As a recently discovered amyloidosis, ALECT2 is critically understudied and under diagnosed. This work is further unique in its ability to discern structures of ALECT2 fibrils from living patient biopsies.

The article is clear and compelling, and, in my opinion, would be suitable for publication provided the following questions were addressed.

The suggestion that a common ALECT2 fibril fold is due to a common cause for their amyloid structure is important, since unambiguous diagnosis would now presumably be accessible via structure determination from biopsies. It would be useful for the authors to further expand on the conditions that would allow this unique characterization of fibrils from live patient biopsies.

In this study, cryo-EM analysis was successful for all five biopsy samples tested, which had been validated by Congo red staining, immunohistochemistry, and immune-EM to confirm the presence of LECT2 aggregation (Fig. 1). Therefore, successful cryo-EM–based amyloid typing requires biopsy samples containing a sufficient abundance of amyloid filaments, which can be preliminarily assessed by Congo red staining. In addition, although amyloid filaments derived from patients with systemic amyloidosis reported to date generally exhibit a clear helical twist, successful helical reconstruction depends on the presence of an apparent helical twist in the extracted filaments. These two points are now included in the manuscript at lines 435-436.

The authors note that two clusters of residues bury charges in the core of the ALECT2 fibrils. How is the protonation state of the residues determined in such a case? In other words, is it certain that the aspartate or glutamate residues in the clusters are not protonated?

As cryo-EM cannot provide direct evidence for protonation, the protonation state of the acidic residues within these clusters is inferred from their local environments. Solid-state NMR studies on β-endorphin filaments have shown that uncompensated carboxyl groups can be protonated, even at neutral pH (PMID: 33046908). In the revised manuscript, we refine the related discussion and propose a coupled filament nucleation–carboxyl protonation model to explain the coexistence of buried uncompensated carboxyl groups and buried salt bridges (lines 374–395).

The authors further suggest that the ALECT2 fibrils may have formed under acidic conditions, implying that the local pKa of some residues is altered in the fibril core. If so, we would expect that altering the pH of the solution containing the fibrils might destabilize them. Is this the case?

We experimentally examined the filament stability of patient-derived ALECT2 filaments under different pH conditions (2.1, 4.3, 7.4, and 9.2 for 24 hours) by negative-stain EM. Filaments were observed under all these conditions, and their morphologies remained largely unchanged, indicating that pH variation has limited impact on the stability of mature filaments. Solid-state NMR studies on β-endorphin filaments have shown that uncompensated carboxyl groups can be protonated even at neutral pH (PMID: 33046908). Therefore, the stability of amyloid filaments under both acidic and neutral conditions may be expected. Extensive filament disassembly was also not observed under alkaline conditions, suggesting that destabilization at two uncompensated carboxyl groups per rung has only a limited effect on the overall filament structure, which is stabilized by multiple inter- and intra-molecular interactions. It is also possible that changes in external pH primarily affect residues at filament ends, as previously discussed for β-endorphin filaments (PMID: 33046908).

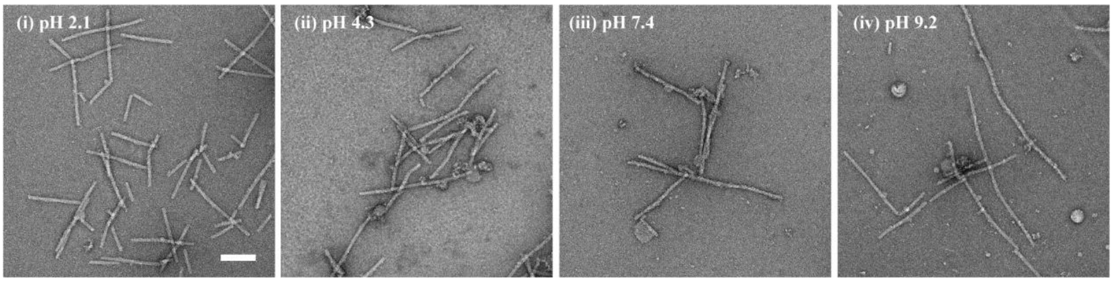

Negative staining micrographs of ALECT2 filaments treated in buffers at different pH (i) pH 2.1; (ii) pH 4.3; (iii) pH 7.4; (iv) pH 9.2 for 24 hours.

The structures determined are of exceptional quality, indicating the identity and geometry of all core residues, and revealing unknown bound densities. While some of those could easily be explained as bound ions or waters, are the authors certain an alternate backbone configuration would not satisfy the oblong density near S11 in their 1a fibril?

Based on the following observations, we are confident that an alternate backbone configuration would not satisfy the oblong density near S11 in type Ia filaments.

1) The oblong density near S11 is clearly separated from the proteinaceous region in our high-resolution cryo-EM maps. If an additional filament subtype with an alternative backbone configuration were present in the

dataset, the oblong density would be expected to be continuous with the protein backbone density in the consensus map.

2) We performed further 3D classification for type Ia filaments, but no additional filament subtypes were present.
3) The oblong density is continuous along the filament axis, whereas proteinaceous density in the amyloid core typically exhibits an axial spacing of ~4.8 Å.

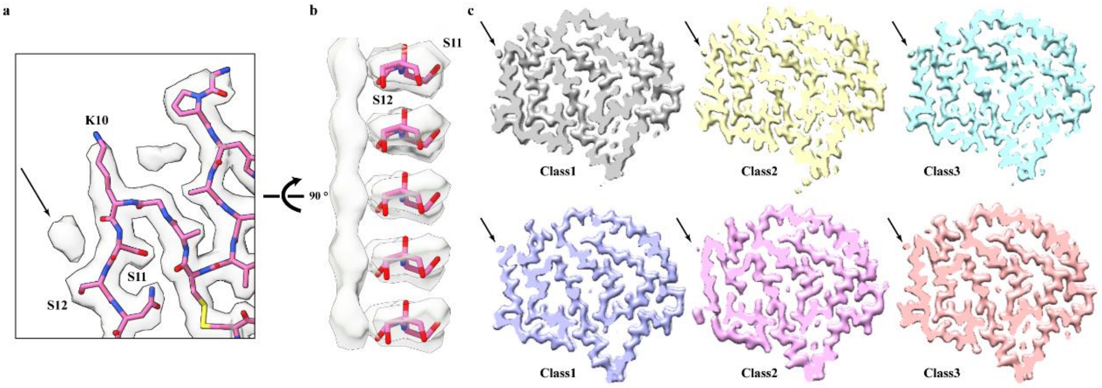

Additional density near S11 in ALECT2 type Ia filaments.
a, **b**, Zoomed-in views of the additional density near S11 in the type Ia filament (black arrow), shown from the top (**a**) and side (**b**). **c**, Class maps from further 3D classification of type Ia filaments.

How do the authors rule out that the paired 1a fibrils are adventitious or that their proportion with respect to unpaired counterparts is a result of purification?

Although we tried to minimize potential disruption to filament structures during extraction, it remains possible that certain steps—such as sonication used to better disperse inclusion bodies—may disrupt paired filaments and thereby affect the relative proportions of paired and unpaired filaments.

In our study, the ratios of paired to unpaired filaments were comparable across all five patient samples examined, despite differences in the extent of sonication applied. Moreover, using *ex vivo* material without sonication, a recent preprint from the Saelices group reported similar proportions of paired and unpaired filaments in a nephrectomy specimen (https://doi.org/10.1101/2025.07.15.664973). Taken together, these observations suggest that the paired-to-unpaired filament ratio is conserved among these ALECT2 amyloidosis patients. This point is now discussed in the revised manuscript (lines 340–344).

The authors indicate several reasons why it is advantageous to determine structures of patient-derived LECT2 fibrils, noting that in vitro generated

fibrils lack the complexity of the former. Can the authors make a similar claim about in vitro grown recombinant LECT2 fibrils seeded with those extracted from biopsies? In other words, are the biopsy-extracted fibrils seed competent?

As suggested, we tested the seeding activity of patient-derived ALECT2 filaments and found that they accelerate the fibril formation of recombinant LECT2 *in vitro*. To facilitate unfolding of native LECT2, the assay was performed at pH 4.4, and fibril formation was monitored by ThT fluorescence at 37 °C with constant orbital shaking. The seeded products formed filamentous assemblies, as confirmed by negative-stain TEM. These results are now included in Supplementary Fig. 1d and 1e, and the corresponding text has been added to the revised manuscript (lines 141-152).

It would be helpful to have some details about the interfaces found in the paired ALECT2 fibrils, for example the shape complementarity and buried surface area per layer, compared to other such interfaces in patient derived amyloid fibrils.

We have expanded the description of the interfaces in the paired ALECT2 filaments, including the buried surface area per rung (lines: 308-312, 322-323) and inter-protofilament interaction analysis (lines 326-328). Interestingly, our analysis shows that the screw-2_1–packed filaments (type IIb) have a larger buried surface area than those packed with C2 symmetry (type IIa), despite their nearly identical cross-sectional structures. This indicates that a half-rise stagger between the two protofilaments enhances the inter-protofilament interactions. In addition, the interface of type II filament is stabilized primarily by side-chain–side-chain interactions, whereas type III filament includes both side-chain–side-chain and side-chain–main-chain interactions, showing higher shape complementarity and being more commonly observed in patient-derived paired amyloid fibrils.

In Supplementary table 2, it would be helpful to note what structures are determined from the same dataset.

The information has now been incorporated into the revised Supplementary Table 2.

Reviewer #3 (Remarks to the Author):

The manuscript reports cryo-EM structures of patient-derived ALECT2 amyloid filaments isolated from patient renal biopsies, describing six-layered protofilament folds with conservation of disulfide bonds. The authors also document minor polymorphisms and dimeric assemblies. Understanding the structures of amyloid fibrils formed by LECT2 is important for establishing disease mechanism, improving diagnostics and ultimately increasing the chance of improved clinical outcomes in the long term. The finding of a shared fold across unrelated cases is interesting and can direct targeting efforts. The authors also report inability to reproduce the patient fribrils with recombinant LECT2 - that is an important finding since in vitro fibrils are usually the material of choice for drug discovery workflows. In my opinion the article merits publication but I have a few comments to be addressed.

The current view of the FSC as reported in Supplementary figure 2 does not allow the reader a proper assessment of overfitting. For the Type Ia and Type Ib, the FSC for the maps do not seem to properly reach zero and just come to a sudden stop. Please report more complete curves for all reconstructions.

We have double-checked the FSC curve and confirmed that all data obtained from a standard "post-process" job in RELION are shown. The raw FSC data have now been included in the Source Data file. A similar FSC curve that does not reach zero has also been reported by the Scheres group (PMID: 33087931, Extended Data Fig. 2f), as shown below. In addition, we contacted the RELION team and received the following reply: "It does not reach 0 simply because there is some signal (albeit very weak) even at the Nyquist frequency. If your pixel size was smaller, it would reach 0 at a higher resolution."

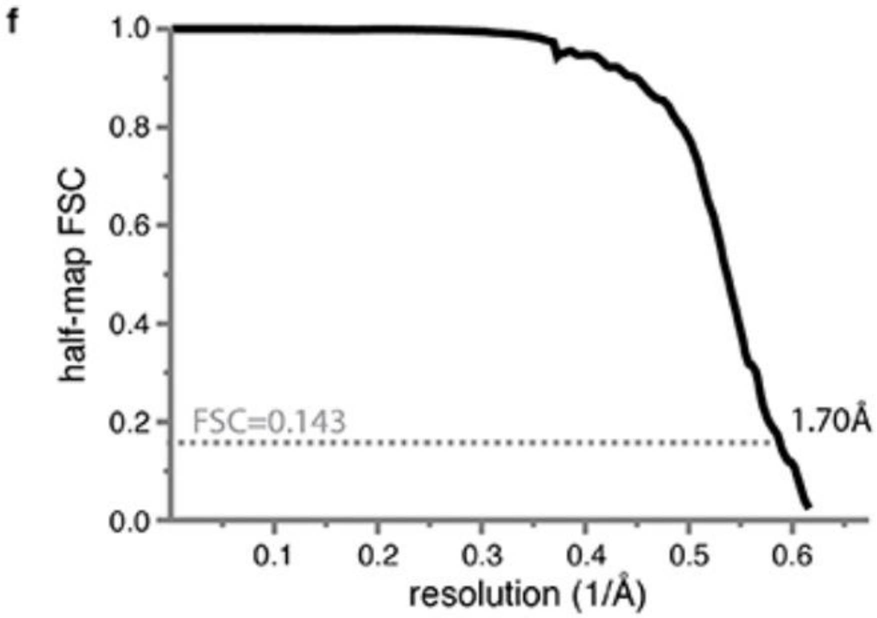

The conservation of the disulfides is quite curious. It would be nice to have a figure highlighting those in a representative fibril and showing the structure of native LECT2 fold and its disulfide bridges for comparison.

Comparison of the local structures surrounding the three disulfide linkages between the native and fibril forms is now included (Supplementary Fig. 4b and lines 225-233).

The manuscript argues that commonly used anti-LECT2 antibodies (presumably raised against the native fold) give inconsistent immunohistochemical detection results because fibrillar ALECT2 incorporates all residues into the ordered cor and that reducies epitope accessibility. Building on your structural insights from this article, could the authors assess or at least speculate whether currently available monoclonal antibodies (that recognize distinct epitopes) could perform better for the detection of the amyloids described in the paper?

Conformation-specific antibodies raised against human-derived filaments would be ideal for detecting amyloid aggregates. However, none of the currently commercial antibodies (listed below) were generated using such filaments as immunogens. Instead, they were raised against either full-length recombinant LECT2 or a peptide spanning residues 48–75. The available information is insufficient to predict how antibodies raised against the full-length protein interact with patient-derived filaments. Based on our structures, residues 48-75 might be a better antigen than the full-length LECT2, as a substantial portion of this peptide (residues 56–75) lies on the

surface of the fibril structure. Nevertheless, the side chains of several residues within this segment (including Q59, N65, N67, N71, and I75) become buried upon filament formation and are therefore not solvent-exposed, which may limit antibody accessibility and binding affinity.

**Clone ID (Product Number)**	**Company**	**Applications**	**Organism**	**Subtype**	**Antigen**
(B-6) sc-398071	UltraCruz	WB，IP，IF，ELISA	Mouse	IgG1	Human LECT2 48-75
(A-7) sc-398072	UltraCruz	WB，IP，IF，ELISA	Mouse	IgG2b	Human LECT2 48-75
(MM0449-4B24) ab89835	abcam	WB	Mouse	IgG1	Full Length human LECT2
(OTI2A11) MA5-25816 ab119429	Thermo Fisher Scientific abcam	WB，IHC	Mouse	IgG1	Full Length human LECT2 in *E.coli*
(LBI2A11) AMM12180V	Annoron (Beijing)	WB，IHC	Mouse	IgG1	Full Length human LECT2 in *E.coli*
(EPR22049-227) ab270338	abcam	sELISA	Rabbit	IgG	/
(3950) 784299ES60	Yeasen	ELISA	Rabbit	IgG	/
UMAF541Hu21	Wuhan Cloud-Clone	WB，IHC，ICC，IP	Mouse	IgG	/
102717 (MAB722)	R&D Systems	IHC, IEM	Mouse	IgG1	Full Length human LECT2 in *E.coli*

Minor point:

40The absence of a fuzzy coat
41 reduces antibody binding to ALECT2 filaments, which may explain the
42 inconsistent immunohistochemical detection and highlight the need for
43 conformation-specific antibodies for accurate diagnosis.f a fuzzy coat
41 reduces antibody binding to ALECT2 filaments, which may explain the
42 inconsistent immunohistochemical detection and highlight the need for
43 conformation-specific antibodies for accurate diagnosis.

I find this sentence confusing. I suggest something along the lines of: “Because all residues are incorporated into the fibril core, epitope accessibility is limited. This can explain variability in immunohistochemical detection and highlight the need for conformation-specific antibodies for improving accuracy in diagnosis”

We have amended these according to your suggestion (lines 40-44).

Reviewer #1 (Remarks to the Author):

The new experiments to characterize the state of disulfide bond formation of the in vitro prepared fibrils are a welcome addition. The data suggest a predominant population of native pairings with some scrambled disulfides present. This finding reinforces the importance of in vivo conditions to LECT2 amyloid structure.

We thank the reviewer for the positive assessment and for the constructive comments provided throughout the review process.

The discussion of the protonation states of buried ionizable groups has been expanded, although I find it still to be speculative and a bit unclear. It would be *less* energetically favorable if D39 and D128 were protonated in their respective salt bridges, as this would leave buried, unsatisfied positive charges from the Lys. Are the authors suggesting that these carboxylates are protonated in the fibrils? For the K110-E122-D125 and K97-E100-C-term charge clusters, protonating both carboxylates in each cluster would be energetically costly for the same reason, so are the authors speculating that only a single carboxylate is protonated in each cluster? There is a lot of hand waving going on without any direct evidence. The authors do state this on lines 391-392, however, so overall this section is probably fine albeit speculative.

We thank the reviewer for the careful evaluation of this section. We agree that, although our interpretation is based on previous studies, the discussion of protonation states remains speculative, as protonation cannot be directly determined from cryo-EM maps at the current resolution.

We believe that the main source of confusion was that our previous description of the selective protonation mechanism was not sufficiently clear. To clarify, we suggest a selective protonation mechanism in which only uncompensated carboxyl groups become protonated, whereas those engaged in salt bridges remain unprotonated. The pK_a values of uncompensated carboxyl groups can increase substantially when buried in a hydrophobic or low-dielectric protein interior. In contrast, carboxyl groups involved in buried salt bridges are stabilized by favorable Coulombic interactions, keeping their pK_a values close to those in aqueous solution despite the surrounding low-dielectric environment (PMID: 25074910).

It now reads:" *In addition, the pK_a values of buried, uncompensated carboxyl groups are known to be substantially elevated relative to their solvent-exposed counterparts, whereas those of buried carboxyl groups forming salt bridges remain near their aqueous pKa values*[41]*. Solid-state*

NMR studies have shown that an uncompensated buried glutamic acid in β-endorphin filaments remains protonated even at pH 7.4[42]. We therefore propose a coupled filament nucleation–carboxyl protonation model: solvent-exposed carboxyl groups with low pK_a values remain unprotonated at the onset of nucleation, enabling salt-bridge formation. As filament assembly proceeds and some of the carboxyl groups become buried within a low-dielectric environment, those stabilized by salt bridges remain largely unaffected, while the pK_a values of uncompensated carboxyl groups increase, favoring their protonation even in the absence of strongly acidic conditions."

The authors addressed the remainder of my questions/concerns well. I recommend this paper for publication in Nature Communications.

Reviewer #2 (Remarks to the Author):

The authors have suitably addressed my comments.

We thank the reviewer for the positive assessment and for the constructive comments provided throughout the review process.

Their revisions raise only one other minor question, regarding the identity of their bound densities: Would a waters placed at those positions be within hydrogen bonding distance of nearby residues? Particularly for the density near S11?

The distance between the extra density (e.g., near S11) and the protein is within hydrogen-bonding range. However, in amyloid filaments, bound water molecules typically appear as discrete, spherical densities with an axial spacing of ~4.8 Å (PMID: 30894745, 34588692). In contrast, the extra densities, especially those near S11, appear continuous along the filament axis rather than as isolated spherical features. We therefore consider it unlikely to represent a water molecule and instead speculate that it may correspond to a non-proteinaceous cofactor, as reported in other amyloid fibrils (PMID: 30894745, 34588692). For comparison, representative densities near residue T17 in the ALCTE type Ia filaments are shown, which are consistent with bound water molecules.

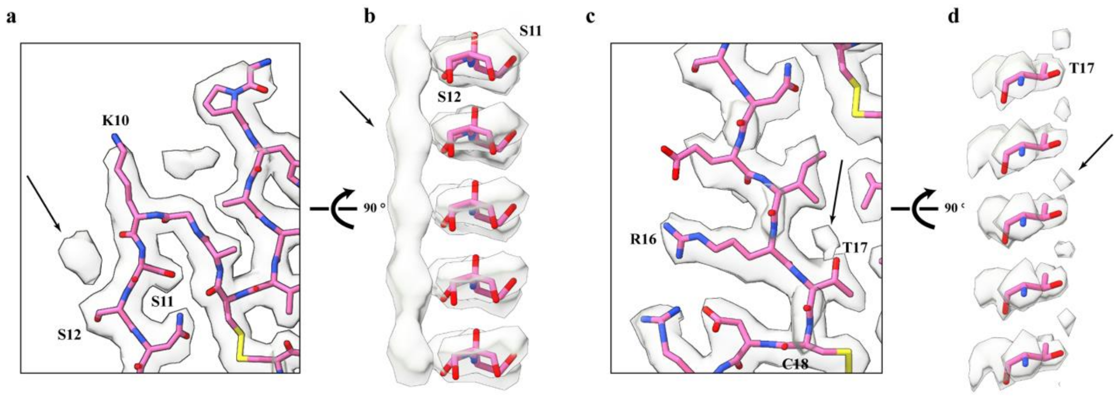

Additional densities in ALECT2 type Ia filaments.
a, **b**, Zoomed-in views of the additional density near S11 in the type Ia filament (black arrow), shown from the top (**a**) and side (**b**).
c, **d**, Zoomed-in views of the additional densities near T17 in the type Ia filament (black arrow), shown from the top (**c**) and side (**d**). These densities are consistent with bound water molecules.

Reviewer #3 (Remarks to the Author):

In my opinion, the authors have addressed the major points of concerns raise by me and the other reviewers. I am happy to recommend the manuscript for publication

We thank the reviewer for the positive assessment and for the constructive comments provided throughout the review process.

Reviewer #1 (Remarks to the Author):

The authors have responded to my concerns adequately.

We sincerely appreciate the constructive feedback provided throughout the review process, which has significantly strengthened our manuscript.

Reviewer #2 (Remarks to the Author):

I find the revisions suitable and appreciate the authors' efforts to improve their manuscript.

We sincerely appreciate the constructive feedback provided throughout the review process, which has significantly strengthened our manuscript.
